# The Isolation and Characterization of a Broad Host Range Bcep22-like Podovirus JC1

**DOI:** 10.3390/v14050938

**Published:** 2022-04-29

**Authors:** Carly M. Davis, Marta K. Ruest, Jamie H. Cole, Jonathan J. Dennis

**Affiliations:** Department of Biological Sciences, University of Alberta, CW 405 Biological Sciences Building, Edmonton, AB T6G 2E9, Canada; davis1@ualberta.ca (C.M.D.); ruest@ualberta.ca (M.K.R.); jhcole@ualberta.ca (J.H.C.)

**Keywords:** *Burkholderia cepacia* complex, bacteriophage, phage, phage therapy

## Abstract

Bacteriophage JC1 is a *Podoviridae* phage with a C1 morphotype, isolated on host strain *Burkholderia cenocepacia* Van1. Phage JC1 is capable of infecting an expansive range of *Burkholderia cepacia* complex (Bcc) species. The JC1 genome exhibits significant similarity and synteny to Bcep22-like phages and to many *Ralstonia* phages. The genome of JC1 was determined to be 61,182 bp in length with a 65.4% G + C content and is predicted to encode 76 proteins and 1 tRNA gene. Unlike the other *Lessieviruses*, JC1 encodes a putative helicase gene in its replication module, and it is in a unique organization not found in previously analyzed phages. The JC1 genome also harbours 3 interesting moron genes, that encode a carbon storage regulator (CsrA), an N-acetyltransferase, and a phosphoadenosine phosphosulfate (PAPS) reductase. JC1 can stably lysogenize its host Van1 and integrates into the 5′ end of the gene *rimO*. This is the first account of stable integration identified for Bcep22-like phages. JC1 has a higher global virulence index at 37 °C than at 30 °C (0.8 and 0.21, respectively); however, infection efficiency and lysogen stability are not affected by a change in temperature, and no observable temperature-sensitive switch between lytic and lysogenic lifestyle appears to exist. Although JC1 can stably lysogenize its host, it possesses some desirable characteristics for use in phage therapy. Phage JC1 has a broad host range and requires the inner core of the bacterial LPS for infection. Bacteria that mutate to evade infection by JC1 may develop a fitness disadvantage as seen in previously characterized LPS mutants lacking inner core.

## 1. Introduction

Antibiotic resistance is a serious global concern, and it is predicted that multi-drug resistant (MDR) bacterial infections will cause over 10 million deaths worldwide annually by 2050 if left unchecked [1]. In 2018 there were just under 1 million bacterial infections in Canada, with a quarter being resistant to frontline antimicrobials [2]. Of these infections, 14,000 people died, and 4 out of 10 deaths would not have occurred if these infections had been susceptible to frontline antimicrobials [2].

Individuals with cystic fibrosis (CF) constantly battle microbial infections and are already experiencing the negative impacts of MDR infections, casting a light on the severity of what lies ahead with MDR infections. *Burkholderia cenocepacia* is a Gram-negative opportunistic pathogen that colonizes the lungs of CF patients. *B. cenocepacia* also has extreme MDR and is associated with poor prognosis and severe patient-to-patient transmission for CF patients [3]. Though infections with *B. cenocepacia* remain relatively low (3.7% of CF patients), they pose a significant threat because of their intrinsic and adaptive resistance to antibiotics and ability to cause life threatening “cepacia syndrome” [3,4,5].

An alternative treatment option for MDR bacteria is the therapeutic use of bacteriophages. Their use as a therapy in North America declined after the discovery of antibiotics, but the increase in antimicrobial resistance has renewed phage therapy as a promising alternative treatment option [6]. Phages are viruses that specifically target and lyse bacterial cells via adsorption to cells using a cellular receptor, injecting their genetic material, and replicating themselves until the cell bursts [6]. Given that the mechanism of action for phages are drastically different from antibiotics, they are an effective tool against MDR bacteria [6]. Additionally, the combined use of antibiotics and phage against bacteria can increase the killing activity of one or both agents, exceeding activity of solo treatment [7,8,9,10]. In this study, we have isolated and characterized JC1, a novel member of the Bcep22-like *Podoviridae* phages. We have shown that JC1 has a broad host range and confirmed the LPS inner core of *B. cenocepacia* strain K56-2 as its receptor. As a first account for this phage group, we have shown JC1 is able to stably lysogenize its host *B. cenocepacia* strain Van1 and that a growth difference exists between wildtype Van1 and the JC1 lysogen.

## 2. Materials and Methods

### 2.1. Bacterial Strains and Growth Conditions

Bacterial strains used in this study are listed in Table 1. *B. cenocepacia* clinical isolate from Vancouver was named Van1 and used for isolation of phages from soil samples. Van1 and the JC1 lysogen were grown aerobically overnight at 37 °C on full-strength Lennox (LB; 10 g/L tryptone, 5 g/L yeast extract, 5 g/L NaCl) solid medium or in LB broth with shaking at 225 RPM unless stated otherwise. All other strains were grown at 30 °C on half-strength LB. Media was supplemented with 50–150 µg/mL tetracycline (Tc) antibiotic for plasmid maintenance when necessary. Suspension media (SM; 50 mM Tris-HCl [pH 7.5], 100 mM NaCl, 10 mM MgSC_4_) was used for all phage work. Any statistical analysis was conducted using GraphPad Prism 9 (Graph-Pad Software Inc., San Diego, CA, USA).

### 2.2. Phage Isolation, Propagation, Host Range Analysis, and Electron Microscopy

Phage JC1 (vB_BceP_JC1) was isolated from potting soil in Edmonton, AB, Canada as previously described [11] with *B. cenocepacia* clinical isolate Van1. Briefly, soil was mixed with 1/2 LB broth, SM, and *B. cenocepacia* Van1 liquid culture and incubated at 30 °C overnight with aeration. The soil slurry was then pelleted by centrifugation and the supernatant was filter sterilized using a Millex-HA 0.45 µM syringe-driven filter unit (Millipore, Billerica, MA, USA). A double agar overlay with Van1 and the supernatant was incubated overnight at 30 °C. A single plaque was isolated using a sterile Pasteur pipette and suspended in 500 µL of SM with 20 µL chloroform to generate a JC1 stock.

Propagation of JC1 was performed at 30 °C using double agar overlays as previously described [11,12], or in liquid. For liquid propagation, 150 µL of a Van1 overnight culture was mixed with 150 µL of JC1 lysate (10^10^ PFU/mL) and incubated for 30 min. After the brief incubation 1.5 mL of SM and 15 mL of LB broth was added to the mixture and incubated overnight with aeration at 225 RPM. Bacterial cells were pelleted by centrifugation at 18,514× *g* for 3 min. The supernatant was collected, and filter sterilized using a Millex-HA 0.45 µM or 0.22 µM syringe-driven filter unit (Millipore, Billerica, MA, USA). JC1 phage stocks were serially diluted into SM and spotted onto soft agar overlays of Van1 to determine stock titer. Plaques were backlit and viewed under magnification using a New Brunswick Scientific colony counter (model C110). Average plaque size was determined from 10 plaques ± standard deviation measured using a digital caliper (Tresna, Guilin, China).

Host range analysis was performed using a collection of 85 phenotypically distinct clinical and environmental isolates listed in Table 1. A high titer JC1 phage stock (1 × 10^10^ PFU/mL) was serially diluted in SM and 5 µL of each dilution was spotted onto double agar overlays of each strain and incubated at 37 °C overnight. Efficiency of plating (EOP) was calculated by dividing the PFU/mL of JC1 on each strain by the actual PFU/mL determined on host Van1. EOP was only calculated for strains that JC1 could produce plaques on. Strains that showed lysis, but no plaques, were included in the host range but were scored using a range of 1 to 3 “+” signs instead of an EOP score.

For electron microscopy, phages were purified by cesium chloride density gradient ultracentrifugation and dialysis. CsCl was dissolved in high titer JC1 lysate to a density of 1.45 g/mL followed by ultracentrifugation at 35,000 RPM in a 50.2 Ti rotor for 20 h at 4 °C. The phage band was extracted into 12 kDa molecular weight cutoff dialysis tubing and dialyzed at 4 °C in 1.5 L SM for 4 days, with the SM buffer changed every 24 h. Ten µL purified phage lysate was loaded onto a carbon-coated copper grid for 2 min and stained with 4% uranyl acetate for 20 s, as previously described [43]. Transmission electron micrographs were captured using a Philips/FEI Morgagni transmission electron microscope with charge-coupled device camera at 80 kV (U. Alberta Dept. of Biological Sciences Advanced Microscopy Facility). The average capsid and tail dimensions ± standard deviation was calculated using Microsoft Excel based on measurements from 10 individual virions taken using ImageJ software (NIH, Bethesda, MD, USA).

### 2.3. Phage DNA Isolation and Sequencing

JC1 genomic DNA (gDNA) was isolated from a high titer phage stock using the Wizard Lambda DNA purification system (Promega Corp., Madison, WI, USA) with some modifications. A 1 mL aliquot of JC1 was incubated with 1 µL of 10 mg/mL DNase I (Thermo Scientific, Waltham, MA, USA), 10 µL DNase I buffer (1 M Tris-HCl, 0.25 M MgCl_2_, 10 mM CaCl_2_), and 0.6 µL of 10 mg/mL RNase A (Thermo Scientific) for 1 h at 37 °C. After incubation, 40 µL of 0.5 M EDTA (pH 8.5) was added to inactivate DNase I and 3.125 µL of 25 mg/mL proteinase K (Applied Biosystems, Carlsbad, CA, USA) and incubated at 55 °C for 1 h to degrade proteins and release phage DNA. The treated lysate was allowed to cool to room temperature and added to 0.84 g of guanidine thiocyanate and 1 mL of pre-warmed (37 °C) resuspended Wizard DNA Clean-Up Resin (Promega Corporation, Madison, WI, USA). This mixture was rocked at room temperature for 20 min, then transferred into a syringe attached to a Wizard Minicolumn (Promega Corporation) and pushed though the column. The column was then washed with 3 mL 80 % isopropanol and dried by centrifugation for 2 min at 10,000× *g*. JC1 phage DNA was then incubated for 1 min with 100 μL of 80 °C sterile milli-Q water (Integrated DNA Technologies, Coralville, IA, USA) and eluted from the column by centrifugation for 1 min at 10,000× *g*. A NanoDrop ND-1000 spectrophotometer (Thermo Scientific, Waltham, MA, USA) was used to determine purity and concentration of eluted DNA. JC1 gDNA was sent for sequencing at The Applied Genomics Core at the University of Alberta. A Nextera XT library prep kit was used to generate the DNA genomic library followed by paired-end sequencing on a MiSeq (Illumina, San Diego, CA, USA) platform using a MiSeq v3 reagent kit. 4.8.

### 2.4. Bioinformatic Analysis of JC1

Read quality was assessed using FastQC v0.11.9 (http://www.bioinformatics.babraham.ac.uk/projects/fastqc/ accessed on 19 January 2022) and trimmed using Trimmomatic v.0.39 with the following parameters: a four-base sliding window that cuts when the average quality per base drops below 20, removes leading and trailing low quality or N bases (below quality 3), and a minimum read length of 35 bp. Of 1,00,666 reads, 92.58% of both read pairs survived trimming and were assembled using SPAdes v3.13.0 [44], resulting in a final contig length of 61,191 bp contig with 1,711,139 reads mapping to 100% of the contig to give an average fold coverage of 3657. PCR and sanger sequencing was used to confirm the assembly by amplifying 13 different regions spanning areas of lower coverage and the “ends” of the contig. The product spanning the ends of the contig lacked a 9 bp repeat sequence, confirming a complete genome length of 61,182 bp. The JC1 genome was determined to be circularly permuted based on read coverage and assembly outputs.

Annotation of the contig was performed using three different annotation software: GLIMMER using the Bacteria and Archaea setting [45], Prodigal [46], and GeneMarkS for phage [47]. BLASTn was used to find related phages and BLASTp was used to identify predicted protein-coding genes and putative functions. Protein-protein BLAST was set to the Bacteria database (taxid:2) when no significant hits were found using the Viruses database (taxid:10239). NCBI non-redundant protein sequence and nucleotide collection databases (update date: 22 January 2022) were used. Hits with an E-value of 1 × 10^−3^ or greater were not considered significant, and annotations were recorded as hypothetical. Conserved domains were identified using Batch CD-Search against the CDD v3.19 58,235 PSSMs database with default settings [48]. TMHMM 2.0 [49], and SignalP 6.0 [50], were used for lysis protein analysis and prediction of lipoproteins, respectively. Aragorn (Galaxy Version 0.6) [51] was used to identify potential tRNA genes. Protein alignments were accomplished using MUSCLE [52]. Whole genome alignments and comparisons were done using MAFFT multiple aligner v1.4.0 [53] and Mauve v1.1.1 [54] plugins for Geneious. The complete genome sequence of JC1 was deposited in GenBank with the accession number OM283127.

### 2.5. Mass Spectrometry Analysis of JC1

JC1 was purified for proteomic analysis using a CsCl density gradient centrifugation. A 150 mL volume of JC1 lysate (10^10^ PFU/mL) was concentrated using ultracentrifugation at 28,700 RPM in a 50.2Ti rotor at 4 °C for 1.2 h. The JC1 pellets were resuspended with SM to a final volume of 16 mL and prepared for CsCl purification according to the manufacturer recommendations (Beckman Coulter 2008). The CsCl gradient was centrifuged at 30,000 RPM in a 50.2Ti rotor at 4 °C for 20 h. JC1 ghost band was removed and dialyzed thoroughly with SM. Phage were prepared for mass spectrometry analysis by boiling purified lysate for 5 min in Laemmli sample buffer (62.5 mM Tris-HCl [pH 6.8], 2% SDS, 10% glycerol, 5% β-mercaptoethanol, 0.001% bromophenol blue), and running on a 10% SDS-PAGE gel. PageRuler Plus Prestained Protein Ladder (Thermo Scientific) was used as a molecular weight standard and 30 µL of JC1 CsCl purified lysate (10^10^ PFU/mL) in 1× sample buffer was loaded into an adjacent well. The lane was excised for whole-lane mass spectrometry analysis at the Alberta Proteomics and Mass Spectrometry (APM) facility located at the University of Alberta. Proteins were considered virion associated if they were identified by two or more unique medium to high quality peptides.

### 2.6. One-Step Growth Curve

One-step growth curve of JC1 on *B. cenocepacia* Van1 was conducted as previously described [7,55] with minor modifications. Overnight liquid cultures of Van1 were subcultured 1:100 and grown for approximately 2 h and 45 min to a CFU/mL of ~3 × 10^7^. JC1 lysate was added at an MOI of approximately 2 and incubated at 37 °C with aeration at 225 RPM. A volume of 10 μL was removed in triplicate every 30 min and immediately serially diluted in 1× PBS. Phage titers were determined by spotting 5 µL of each dilution on soft agar overlays containing Van1 culture. Burst size was calculated using the formula “burst size = P/I” where P is the maximum number of phages after lysis and I is the number of phages initially added to the culture. Resulting data from three replicates were analyzed using GraphPad Prism 9 (GraphPad Software Inc., San Diego, CA, USA).

### 2.7. Complementation of LPS Mutants

Plasmids used in this study are listed in Supplementary Table S1. The LPS genes for each of the seven mutants were amplified from *B. cenocepacia* K56-2 gDNA using primer pairs listed in Supplementary Table S2. Resulting PCR products were digested with XbaI and KpnI Fast Digest restriction endonucleases (Thermo Scientific), ligated with T4 DNA ligase (NEB) into the vector pSCrhaB2-Tc, and transformed into *Escherichia coli* DH5α. Each resulting construct was verified with Sanger sequencing and transformed into the desired electrocompetent K56-2 LPS mutant strain.

### 2.8. Identification of Phage Receptor

High titer JC1 (10^10^ PFU/mL) was spotted onto double agar overlays of wt K56-2 or LPS mutants carrying either an empty vector control or the complementation plasmid and observed for lysis. Receptor analysis was examined further as previously described [56]. Phage adsorption assays were performed with *B. cenocepacia* Van1 and K56-2 cultures treated with either periodate or proteinase K to destroy either LPS or cell surface proteins, respectively. For proteinase K treatment, 2 mL of culture was treated with 0.2 mg/mL proteinase K (Applied Biosystems, Carlsbad, CA, USA), incubated at 37 °C for 3 h, and washed 2× with LB. For periodate treatment, 2 mL of culture was centrifuged at 6000× *g* for 3 min, and the bacterial pellet was resuspended in 1.5 mL sodium acetate (50 mM; pH 5.2) or sodium acetate with 10 or 100 mM IO_4_^−^ and incubated for 2 h (protected from light). The cells were then washed 2× with LB. Bacterial suspensions were standardized using OD_600_. A 100 µL volume of JC1 (1 × 10^6^ PFU/mL) was incubated with a 500 µL sample of each treated bacteria, as well as an LB negative control and an untreated bacterial control for 30 min at room temperature. These samples were then centrifuged at 13,523× *g* for 3 min and tittered to determine the PFU/mL. The phage titer in the negative control supernatants were set to 100%. Each assay was performed in technical and biological triplicate.

### 2.9. Determination of JC1 Lifestyle and Integration Site

A liquid propagation of JC1 and Van1 was set up as described above. Surviving cells were collected by centrifugation at 6000× *g* for 5 min, and the supernatant was discarded. Cells were washed 3× with LB broth to remove any extracellular phage and then resuspended in 5 mL LB broth. The washed cells were incubated again overnight at 37 °C with aeration at 225 RPM. The culture was then streaked onto LB solid media to obtain single colony isolates. Single colonies were then tested for superinfection resistance using overnight cultures of every isolate in a top agar overlay assay with Van1 spotted on top. Plates were incubated overnight at 37 °C and observed for zones of lysis. Colony PCR was also performed on each single colony to detect the presence or absence of the JC1 genome. Single colonies with no zone of lysis and a positive PCR result were retained for further analysis.

Stability was analyzed by streaking out 4 different JC1 lysogen isolates on to LB agar plates and incubating for 2 days at 37 °C. A single colony was picked from these plates and struck out onto a new plate to obtain a second streak out. This was then done a third time to obtain a third streak out. One colony from each streaked plate for each lysogen was tested for superinfection resistance to JC1 and the presence of the JC1 genome using PCR as described above.

Determination of JC1 integration site was conducted as previously described [57] with some modifications. Primers were made that flanked the 133 bp intergenic region upstream of the predicted serine recombinase (gp1) (Supplementary Table S2). Genomic DNA from a confirmed lysogen and pUCP22 were digested with SalI and ligated overnight with T4 DNA ligase (New England Biolabs) at 4 °C. 2.5 µL of the ligation mix was then used as a template in PCR using combinations of the primers that flank the upstream region of gp1 and M13 primers that flank the MCS of pUCP22. PCR products were sent for Sanger sequencing and analyzed using BLAST (https://blast.ncbi.nlm.nih.gov/Blast.cgi). The integration site was further confirmed with PCR using primer pairs that anneal to the Van1 genome flanking the identified integration site.

### 2.10. Growth Analysis of Van1 vs. JC1 Lysogen and JC1 Virulence Index

Potential growth differences between *B. cenocepacia* Van1 and JC1 lysogen were assessed in 3 complete medias: LB broth, Mueller Hinton (MH) broth, and tryptic soy broth (TSB). Overnight cultures of each strain were subcultured 1:100 in LB broth and incubated at 37 °C for 2 h and 45 min. Subcultures were further diluted 1:100 into their desired media to a final CFU/mL of 1 × 10^6^. A volume of 200 µL was added to each well of a 96-well plate and placed in an Epoch™ 2 Microplate Spectrophotometer (BioTek) at 37 °C with orbital shaking at 237 cpm. OD_600_ was measured every hour for 48 h. Growth rate was calculated for exponential growth with the averages from each trial using growth rate equation:

log_10_N − log_10_N_0_ = (μ/2.303) · (t − t_0_) (1)

The JC1 kill curves were performed similarly as described above with modifications. Van1 was prepared in LB as described above and 100 µL was mixed with 100 µL of JC1 diluted in LB to reach each desired multiplicity of infection (MOI). The 96 well plate was measured in an Epoch™ 2 Microplate Spectrophotometer (BioTek) at 30 °C or 37 °C with orbital shaking at 237 cpm. OD_600_ was measured every hour for 48 h. Local virulence was calculated by dividing the area under the curve for each MOI by the area under the curve of the bacterial control and subtracting that from 1. Global virulence index was calculated by taking the area under the curve generated from plotting the local virulence at each MOI tested against log_10_ MOI and diving it by 6 (all the MOIs tested-1). It is important to emphasize that global virulence indexes can only be compared if MOIs tested in the experiment are the same. Results for growth and kill curves were collected in technical and biological triplicate and analyzed using GraphPad Prism 9 (GraphPad Software Inc., San Diego, CA, USA).

### 2.11. Virulence Assay Using Galleria mellonella

*G. mellonella* infections were performed as previously described [58] with some modifications. Overnight cultures of each strain were washed 3× with 1× PBS and serially diluted tenfold in 1× PBS. Scientific grade *G. mellonella* weighing between 300–320 mg were purchased from Serum Therapeutics Inc. (https://www.serumtherapeuticsinc.com/) and injected with 5 µL of bacterial culture or 1× PBS into the rear left proleg using a 250 µL Hamilton syringe. Injection with sterile 1× PBS was used as a negative control to show larvae were not dying because of injection. Ten larvae were injected for each group and statically incubated at 37 °C in the dark. Colony counts were used to determine the CFUs injected. Larvae were scored for death based off response to gentle touch every 24 h until 96 h post infection. Resulting data from three separate trials were plotted and analyzed using GraphPad Prism 9 (GraphPad Software Inc., San Diego, CA, USA).

### 2.12. Normal Human Serum (NHS) Percent Survival

Percent survival in NHS was based on previously published methods [59,60] with modifications. Overnight liquid cultures of Van1 and Van1::JC1 lysogen were subcultured 1:100 and grown for approximately 3 h and 15 min in LB at 37 °C with aeration at 225 RPM. 2 μL of each subculture (10^4^–10^5^ CFU) was inoculated into 198 μL of pooled normal human serum (NHS) purchased from BioIVT (Westbury, NY, USA) diluted in LB for final concentrations of 0–100% NHS. Inoculated NHS was statically incubated at 37 °C with humidity for 2 h followed by serial dilution to obtain CFU counts. The resulting data from three separate trials was normalized to the 0% NHS control and was analyzed using GraphPad Prism 9 (GraphPad Software Inc., San Diego, CA, USA).

## 3. Results and Discussion

### 3.1. Isolation, Morphology, and Host Range

**Figure 1:**
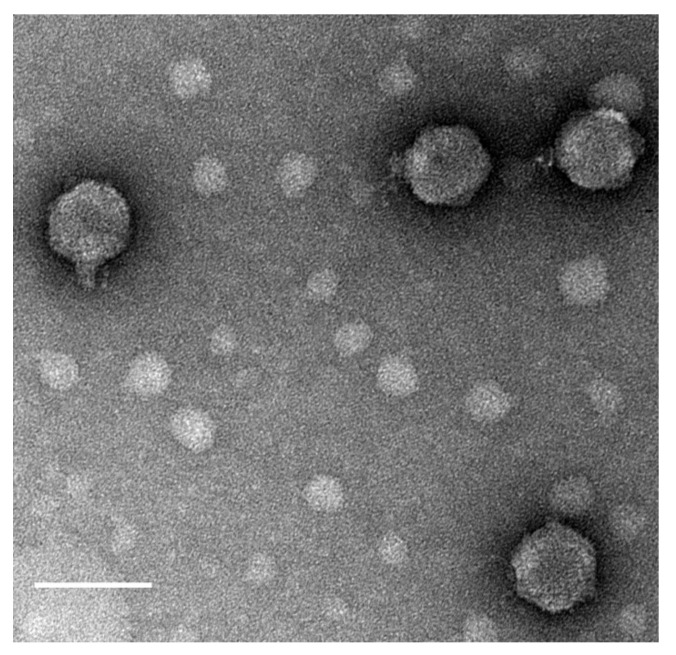
Transmission electron micrograph of JC1. High titer CsCl gradient purified JC1 virions were stained with 4% uranyl acetate on a copper grid and viewed at 140,000× magnification with a transmission electron microscope. Measurements of 10 phage particles have an average capsid diameter of 71 nm ± 1.24 nm and a short, noncontractile tail measuring 20 nm ± 0.91 nm in length and 13 nm ± 0.67 nm in width. Scale bar represents 100 nm.

*Burkholderia* phage JC1 (vB_BceP_JC1) was isolated from potting soil containing geranium (*Geranium dissectum*) and petunia (*Petunia exserta*) annual flowers using cystic fibrosis clinical isolate Van1. JC1 produces clear plaques with a diameter of 1 to 2 mm with overnight incubation at 37 °C and forms slightly turbid plaques of the same size at 30 °C after a 2-day incubation. Transmission electron microscopy (TEM) was used to visualize JC1 and classify it as a *Podoviridae* phage with a C1 morphotype [61], having an average capsid diameter of 71 nm ± 1.24 nm and a short non-contractile tail with a length of 20 nm ± 0.91 nm and a width of 13 nm ± 0.67 nm. (Figure 1).

**Figure 2:**
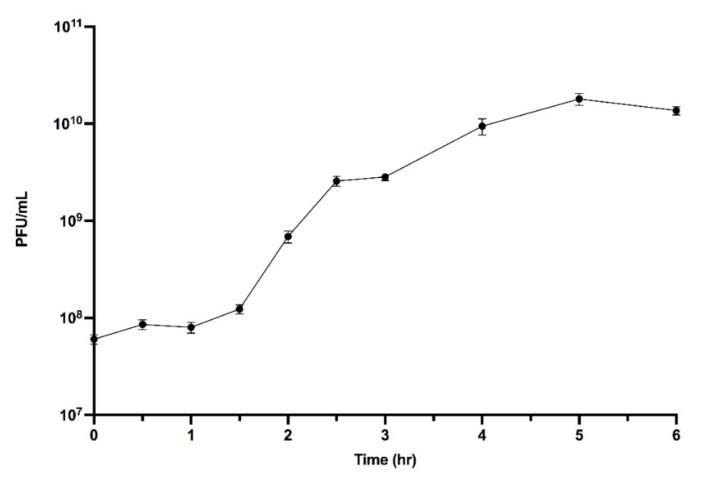
JC1 one-step growth curve on *B. cenocepacia* strain Van1. Subcultured Van1 was grown to approximately 3 × 10^7^ CFU/mL at 37 °C. JC1 lysate was added at an MOI of ~2 and incubated at 37 °C with aeration at 225 RPM. Samples were taken every 30 min for 3 h, followed by every hour for 3 h, and serially diluted in 1× PBS. A total of 5 μL of each dilution was spotted on soft agar overlays containing Van1. Error bars represent the standard error of the mean (SEM). Data from three biological replicates is shown. Phage JC1 exhibits a latent period of 1 h and 30 min and a burst size of 296 virions per cell at 6 h.

Tail fibers were not observable in the TEM images. JC1 morphology is similar to Bcep22, BcepIL01, and DC1 [62,63], suggesting it may be a member of the Bcep22-like phage group. A one-step growth curve of JC1 on host strain Van1 shows a latent period of 1.5 h and a burst size of 296 virions at 6 h (Figure 2).

The host range of JC1 was performed on a large panel of 85 Bcc clinical and environmental isolates revealing a very broad host range. JC1 is capable of infecting an impressive range of *Burkholderia* species including *B. cepacia*, *B. multivorans*, *B. cenocepacia*, *B. stabilis*, *B. vietnamiensis*, *B. dolsa*, *B. ambifaria*, *B. anthina*, Bcc Group K, *Burkholderia* sp., and *Ralstonia pickettii*, which possesses high similarity to Bcc [64]. JC1 showed lytic activity against 50 of the 85 strains, successfully forming plaques on 29 of the 50 (Table 1).

### 3.2. Receptor Binding

**Figure 3:**
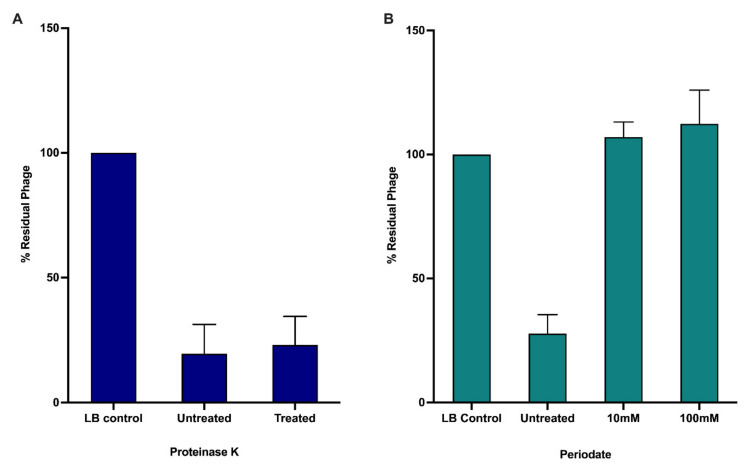
Effects of proteinase K and periodate treatment on JC1 adsorption to *B. cenocepacia* Van1. Bacterial overnights were incubated with either (**A**) proteinase K or (**B**) or periodate to observe if JC1 can adsorb to bacteria without surface proteins or carbohydrates, respectively. Error bars represent the standard error of the mean (SEM). Data from three biological replicates is shown.

A significant number of *Burkholderia* phages likely use the LPS as their primary receptor for infection [65]. A collection of *B. cenocepacia* K56-2 LPS mutants have been previously constructed and characterized [66,67]. Plasmids complementing each LPS mutant were constructed and transformed into their designated strain. The collection of 7 LPS truncation mutants and their complemented strains were screened to determine if JC1 uses the LPS as its receptor. JC1 can infect K56-2 lacking an O-antigen and the outer core but is unable to infect mutants lacking varying degrees of the inner core (Table 2) [66,67]. Complementation of the three LPS truncation mutants restores JC1 infection. Since LPS make up a significant proportion of the outer membrane of Gram-negative bacteria, extreme truncations of the LPS may affect the organization of the membrane and may be indirectly affecting the ability of JC1 to infect SAL1, CCB1, and XOA8 [67]. To further investigate if the LPS is the primary receptor of JC1, we examined phage adsorption against Van1 treated with either proteinase K or periodate, which destroy surface proteins or carbohydrates, respectively. JC1 was able to adsorb to untreated and proteinase K treated cells, but JC1 was unable to adsorb to cells treated with periodate (Figure 3). These results paired with the screening of the LPS mutants confirm that LPS is the primary receptor for JC1.

Truncation of the LPS to evade infection by JC1, or other phages that require the inner core for infection, should increase sensitivity to antimicrobial peptides such as polymyxin B, melittin, and human neutrophil peptide 1 (HNP-1) and possibly affect survival in vivo as seen in previous work characterizing strains lacking a complete LPS core oligosaccharide [66,67]. Combination therapy with these antimicrobials and phage could therefore prove very effective at reducing resistance and increasing sensitivity to both killing agents.

### 3.3. Genomic Characterization

**Figure 4:**
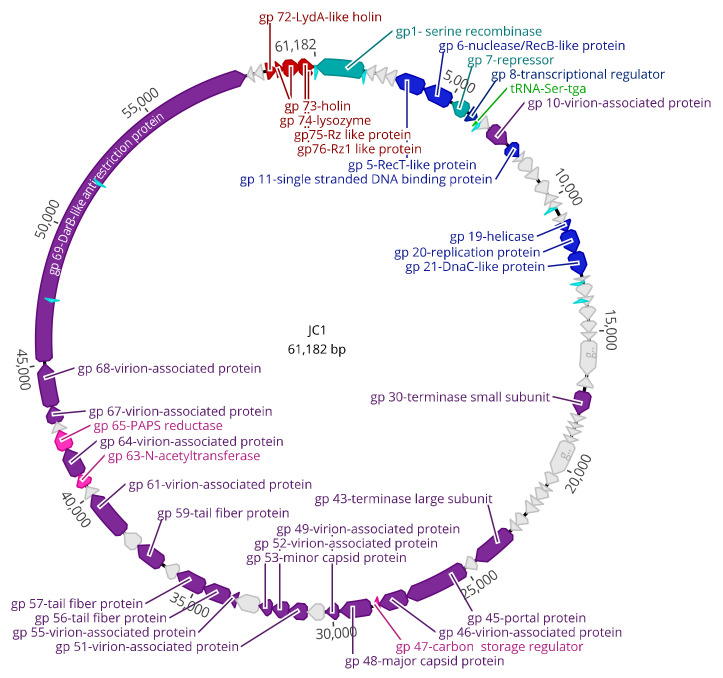
Circularized genomic map of JC1. Scale (in bp) is shown on the outer periphery. Assigned putative functions for each of the 76 predicted open reading frames are as follows: lysis (red), DNA replication, repair, and regulation (blue), lysogeny (teal), virion morphogenesis (purple), hypothetical (grey), tRNA (green), moron (pink), Rho-independent terminator (light blue). JC1 has a GC content of 65%. Image created using Geneious Prime.

**Figure 5:**
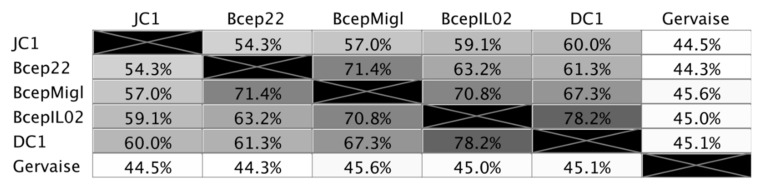
Percent identity of Bcep22-like podoviruses and *Ralstonia* phage. Multiple sequence alignment was performed using MAFFT. Identical nucleotides between each genome is represented in greyscale and percent.

The genome of JC1 is 61,182 bp in length (Figure 4) with a 65.4% G + C content and a coding density of approximately 95%. BLASTn analysis of the JC1 genome shows it is related to the Bcep22-like *Podoviridae* phages and belongs to the *Lessievirus* genus. JC1 is most similar to DC1 with 90.88% identity over 61% of the genome. Interestingly, JC1 also has similarity to a number of *Ralstonia* phages, which likely explains why JC1 is able to infect *R. picketti*. Using MAFFT alignment, JC1 is the most divergent of the Bcep22-like phages, with 54.3%, 57%, 59.1%, and 60% identity across the whole genome to Bcep22, BcepMigl, BcepIL02, and DC1, respectively (Figure 5).

**Figure 6:**
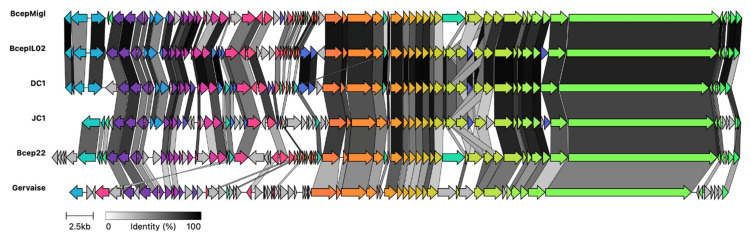
Clinker gene cluster comparison of Bcep22-like phages and *Ralstonia* phage Gervaise. Comparison of whole genomes for *Burkholderia* phage JC1 against the four other Lessievirus phages DC1, BcepIL02, Bcep22, and BcepMigl and related *Ralstonia* phage Gervaise. Percent amino acid identity is represented by greyscale links between genomes. Homologous proteins are assigned a unique color.

Though ~40% of the JC1 genome is dissimilar to the other Bcep22-like phages, and ~55% of the *Ralstonia* phage Gervaise differs from each Bcep22-like podovirus, the retained synteny between the phage genomes is apparent (Figure 6). Each phage encodes a putative serine or tyrosine recombinase on the reverse strand, a repressor-like gene, as well as a serine tRNA. Their entire, or almost entire, virion morphogenesis and lysis modules are encoded on the positive strands. Each phage encodes 3–4 tail fiber proteins followed by a conserved protein annotated as the head closure protein in *Ralstonia* phages. Additionally, all 6 of the genomes encode a massive DarB-like protein on the positive strand followed by two hypothetical proteins on the negative strand and the lysis module. Bcep22 and DC1 have the highest G + C content of the *Lessieviruses* at 66.2% while BcepIL02, BcepMigl, and JC1 have a lower G + C content around 65%, below that of *B. cenocepacia,* which possesses a 66.9% G + C content [68]. Noticeably, JC1, Bcep22, and BcepMigl all lack the presence of the PagP-like virulence factor found in BcepIL02 and DC1 [62,63]. Similarly to Bcep22 and BcepIL02, JC1 appears to be a circularly permuted phage, and the genome was set to begin after the predicted lysis module [62].

There are 76 predicted protein-coding genes and one predicted serine tRNA gene (Table 3, Figure 4). The predicted gene products have 12 GTG start codons and 64 ATG start codons. There are 50 TGA stop codons, with the remainder being 21 TAA codons and 5 TAG codons. BLASTp analysis of the 76 predicted proteins identified significant matches for every predicted gene product in the genome (Table 3, Figure 4). Predicted proteins gp4, gp16, gp17, gp33, gp34, gp37, gp39, gp42, and gp66 had no significant hits to the viruses database, but had hits to the bacterial database, all of which had very high percent identity to *B. multivorans* strains AU34603 (JAHPNN010000013.1), AU36904 (JAHPNA010000002.1), AU9032 (JAHPOS010000011.1), and AU11550 (JAHPOP010000012.1). Upon further analysis of these incomplete genomes, it appears that a potential 6^th^ member of this podovirus group exists stably integrated into all four of these strains, though functionality of the prophage is unknown. It is important to note some base pair differences exist between the 4 lysogens that likely arose overtime from integration in different strains. This prophage harbours around 96.4% similarity to JC1 and has all the genomic similarities discussed above with the other Bcep22-like phages.

Rho-independent termination sites were predicted using ARNold [69,70,71,72]. The 8 predicted sites are displayed in Table 4 and are located downstream of gp2 (hypothetical protein) and downstream of the serine tRNA, upstream of gp17 (hypothetical protein), downstream of gp22 (hypothetical protein), one within the coding region in the opposite direction of gp24 (hypothetical protein), two within the coding region in the opposite direction of gp69 (DarB-like antirestriction protein), and one immediately downstream of the lysis module (gp72–76).

### 3.4. DNA Replication, Repair, and Regulation Module

JC1 has at least 7 proteins involved in DNA replication, repair, and regulation spanning genes 5 through 21 (Table 3, Figure 4). Putative functions determined via BLASTp include RecT/RecB (gp 5/6), which may aid in phage recombination events, a transcriptional regulator (gp8), a single stranded DNA binding protein (gp11), a helicase (gp19), a replication initiator protein (gp20), and a DnaC-like helicase loader (gp21) (Table 3, Figure 4). An interesting hypothetical protein in this module is gp18, which is present in each Bcep22-like phage at the beginning of the gene cluster containing the replication initiator protein and the DnaC-like helicase loading protein. This protein has a predicted helix-turn-helix domain and likely binds to DNA (Table 5); though its function is unknown, the conserved sequence location and high percent identity (>77% to each homolog) suggests it may have an important role in DNA replication.

JC1 follows a trend observed in Gram-negative phages where the recombination genes (gp5/6) are located between the repressor and the integrase genes (gp1/gp7) [73]. Interestingly, unlike Bcep22, BcepIL02, DC1, and BcepMigl, JC1 encodes a putative helicase protein (gp19) predicted both by BLASTp and conserved domain search results (Table 3 and Table 5). Furthermore, it does not follow the typical organization of an initiator-helicase loader-helicase (ILH-type) replication module seen in other phage, where the helicase protein is downstream of the replication initiator protein and the helicase loading protein [73]. Instead, the helicase protein is encoded upstream of the initiator protein, making it a helicase-initiator-helicase loader (HIL) replication module. This organization is also seen in the *B. multivorans* prophage discussed above, but whether this is a common module organization or unique to these phages is unknown and requires an in-depth evaluation of other phage genomes.

### 3.5. Virion Morphogenesis Module

The virion morphogenesis module takes up over half of the genome, is composed of at least 19 predicted proteins spanning from gp30 to gp69, and is encoded entirely on the positive strand (Figure 4). BLASTp, conserved domain search, and homology to other phages were used to putatively assign functions for these proteins and include a small and large terminase subunit (gp30 and gp43, respectively), a portal protein (gp45), a major capsid protein (gp48), three virion associated proteins (gp49, gp53, gp55, and gp67), three tail fiber proteins (gp56, gp57, gp59), a head closure protein (gp61), and a DarB-like antirestriction protein (gp69) (Table 3 and Table 5). The end of this module is marked by two hypothetical genes on the reverse strand, and as discussed above, are highly conserved among the Bcep22-like phages.

Similar to Bcep22 and BcepIl02, the terminase small subunit is located a significant distance away from the terminase large subunit, contrasting what is seen in lambdoid phage and many other Bcc phage genomes [63,74,75,76,77,78,79,80]. BcepB1A is a *Myoviridae* phage that displays a degree of mosaicism to the *Lessievirus* phages and exhibits the larger distance between the two terminase subunits [81], suggesting this organization is not distinct to *Lessieviruses*. Conclusive with comparisons done by Gill et al. [62], the terminase large subunit in JC1 is also related to the *terL* homologs in *Pseudomonas aeruginosa* phage F116 (YP_164303.1) and *Sinorhizobium* phage PBC5 (YP_010115347.1), and the putative portal protein, major capsid protein, and head closure protein all have homology to *E. coli* phage 933W (NP_049512.1, NP_049514.1, NP_049522.1). These genomic similarities paired with JC1 being a terminally redundant circularly permuted genome suggests that phage in this group likely package their genomes via a headful mechanism.

The DarB-like antirestriction protein takes up a massive portion of the virion morphogenesis module (32.7%) and 22.1% of the whole phage genome. This protein is most similar to the DarB homolog in Bcep22 (gp75) and is also commonly found in many *Ralstonia* phages. *E. coli* phage P1 requires virion-associated proteins DarA and DarB to protect P1 DNA from restriction by the host type I restriction system [82], and as discussed previously likely provide a significant benefit to the Bcep22-like phages given the extra burden the size of these genes impose [62]. Interestingly, the P1 antirestriction system has been shown to require additional proteins including DdrA, DdrB, Hdf, and Ulx [83]. No homologs of any of these other proteins have been identified in the *Lessieviruses* thus far, however there are a significant number of virion-associated proteins with no known function and therefore it is likely homologs of these genes exist.

### 3.6. Lysis Module

The lysis module is a collection of 5 genes (gp72–gp76); surprising given the lysis modules of the other four phages contain the typical SRRzRz1 lysis organization [62,63,84]. The module begins with two predicted holin genes and a lysozyme (gp72–gp74). The LydA-like holin (gp72) has a conserved Phage_holin_3_3 domain, and TMHMM predicts the protein to have two transmembrane domains, classifying it as a superfamily III, family 34 holin [85]. The stop codon of gp72 overlaps with the start codon of gp73, similar to how *lydA* and *lydB* are organized in coliphage P1 [86]. However, unlike P1 LydB, gp73 is predicted to have two transmembrane domains. This is unusual as antiholins of class I and II holins typically display a dual-start motif or have been seen to be coded completely within the holin gene [87,88]. With that said, many *Streptococcus* phage encode a class I and class II holin (respectively) upstream of an endolysin, and it is likely the second holin gene acts as an antiholin [89]. The third gene in this module is a predicted lysozyme (gp74) with a conserved ZliS superfamily domain. These enzymes hydrolyze the β1,4-glycosidic bond in peptidoglycan, and gp72–gp74 are found in a similar organization as some Type X Secretion Systems (TXSS): two holins preceding a ZliS superfamily muramidase [90,91].

The top BLASTp hits for gp72–gp74 do not involve any *Burkholderia Podoviridae* phages. The LydA-like holin (gp72) is homologous to uncultured *Caudovirales* phages, and *Siphoviridae* phages from *Nitratiruptor*, *Psychrobacter*, and *Moraxella* species. Similarly, the second holin gene (gp73) is most related to *Siphoviridae* phage AH2 and *Myoviridae* phage PE067. Continuing the trend, the top BLASTp hit for the lysozyme (gp74) is to a *Rhodoferax* podovirus, and the rest of the top hits are to *Siphoviridae* phages. The overall identity is on the lower end, ranging between 50–65% over 80 to 95% of the query, and the evolutionary origin of these three genes is unknown.

The lysis module of JC1 is homologous to the two spanin subunits found in all the other *Lessievirus* phages. The Rz and Rz1 proteins (gp75 and gp76, respectively) also belong to the embedded class of Rz1 genes, where the entire coding sequence of Rz1 is found within the coding sequence of Rz [62,84]. Rz (gp75) is predicted to have an N-terminal transmembrane domain (TMD) and SignalP analysis of Rz1 (gp76) predicts a lipoprotein signal peptide (Sec/SPII) with a cleavage site between amino acids 19 and 20, resulting in a final processed protein of 54 amino acids [48,49].

The lysis module of JC1 differs in the mode of lysis from the rest of the phages in the *Lessieviruses* genus, an interesting feature considering how similar the genomes are to one another. The other *Lessieviruses* likely lyse cells using pinholins and SAR endolysins [62], while JC1 likely lyses cells using the canonical holin mechanism. Though Lynch et al. [63] has predicted gp68 in DC1 (homolog of gp70 in JC1) to be a putative antiholin based on the TMHMM prediction of a single transmembrane domain (also predicted in all the *Lessieviruses* homologues), it is likely this protein plays a different role after analysis of the JC1 lysis module. It has been suggested that pinholins are an intermediate stage in the evolution of holin-endolysin systems, with the canonical holins having a selective advantage [92]. Therefore, this mechanistic difference in lysis may potentially play a role in the larger host range exhibited by JC1, but further studies are needed to examine the lysis potential of these modules.

### 3.7. Moron Genes

The genome of JC1 harbours three interesting moron genes, a carbon storage regulator (CsrA), an N-acetyltransferase, and a phosphoadenosine phosphosulfate (PAPS) reductase (gp47, gp63, and gp65, respectively). All three of these genes are present in the *Lessieviruses*, in similar locations and with high percent identity between homologues. CsrA has been associated with a massive array of functions in bacteria, including but not limited to carbon metabolism, virulence, motility, and biofilm formation [93]. Gp63 has a conserved RimL domain, involved in acetylating the ribosomal L12 protein [94]. N-acetyltransferases have been proposed to be evolutionary precursors of the eukaryotic histone acetyltransferases [95], and therefore the N-acetyltransferase encoded by JC1 may play a role in modifying gene regulation. PAPS reductases are a class of sulfonucleotide reductases (SRs) that are involved in catalyzing the reduction of adenylated sulfate to sulfite, a crucial step in the cysteine biosynthesis pathway [96]. PAPS reductases are found in pathogenic bacteria such as *E. coli*, *Salmonella typhimurium*, and *Yersinia pestis* and are not found in humans, making this class of protein interesting as potential targets for therapeutic intervention [96]. The JC1 PAPS reductase (gp65) likely plays a role in sulfate reduction and could play a role in pathogenicity. Further experiments are needed to investigate the functions these moron genes play in the infection cycle of JC1 and the other *Lessieviruses*.

### 3.8. Analysis of JC1 Structural Proteins

We performed proteomic analysis on CsCl purified virions to identify any unknown virion-associated proteins and confirm predicted virion-associated proteins. Proteins determined to be virion-associated are listed in Table 6 and include the portal protein (gp45), the carbon storage regulator (CsrA) (gp47), the major capsid protein (gp48), the head closure protein (gp61), four virion-associated proteins (gp49, gp53, gp55, and gp67), six hypothetical proteins (gp10, gp46, gp51, gp52, gp64, and gp68), and the DarB-like antirestriction protein (gp69). As expected, the most abundant protein identified was the major capsid protein. The six hypothetical proteins have been assigned putative functions as virion-associated proteins in Table 3, though their functions beyond that are unknown.

Interestingly, gp62 did not show up in the mass spectrometry data even though it is predicted to be virion associated. Mass spectrometry analysis done by Gill et al. [62] did not identify gp68 in Bcep22 (a homolog of JC1 gp62) as virion-associated, but it was predicted to be virion-associated because the homolog was identified in BcepIl02 (gp64). Given that this protein was not identified by mass spectrometry for Bcep22 or JC1, it seems likely that this protein is not virion-associated for these two phages. Reasoning for the exclusion of this protein in Bcep22 and JC1 is speculative but could be associated with the number of tail fiber genes each phage encodes, as BcepIL02 has 4 tail fiber genes whereas JC1 and Bcep22 have three. Mass spectrometry analysis would need to be conducted on DC1 and BcepMigl for further insights.

The carbon storage regulator protein (CsrA) was identified by mass spectrometry, but only one unique peptide was associated with it, and therefore though it is likely to be virion associated it cannot be definitively confirmed. No proteins were identified when the spectrometry data was screened against the UniProt *Burkholderia* database, suggesting this protein is not a result of bacterial protein contamination. Inclusion of CsrA in the actual virion is an interesting and exciting possibility, showing that it is not only a moron gene, but is providing an unknown benefit to the phage. Further experiments are necessary to explore the role of CsrA in infection, and if it is virion-associated in the other *Lessieviruses*.

An unexpected finding is that the hypothetical protein gp10 is virion associated. This protein has a conserved DUF2303 superfamily domain; homologs of this gene are in BcepIL02, BcepMigl, and DC1, but no homolog was found in Bcep22. The predicted structure of gp10 using Phyre2 shows similarity to a viral genome injection device from *Lactococcus* phage TP901-1 (90.4% confidence, 16% identity), a cytosolic disulfide reductase (DsbM) from *Pseudomonas aeruginosa* (49.3% confidence, 16% identity), and a *Citrobacter* restriction-modification controller protein (38.4% confidence, 20% identity). Given that gp10 is located in the replication and repair module, it is possible that this protein is associating with the DNA and not the actual structure of the virion. Gp10 could be similar in function to gp2 in T4 coliphage, associating with the ends of the DNA and protecting it from exonuclease activity [97,98].

### 3.9. Integration Site Characterization

The presence of lysogeny genes gp1 and gp7 led us to examine the potential of JC1 to form lysogens in Van1. Phage infection survivors that were superinfection resistant and had the presence of the JC1 genome verified using PCR were collected for further examination. Of the four previously characterized Bcc *Podoviridae* phages, one is obligately lytic and the others form unstable lysogens in the bacterial hosts tested [62,63,77]. Stability was tested and showed that lysogens maintained superinfection resistance and the presence of JC1 genome after three sequential platings, suggesting JC1 can stably lysogenize Van1. Genomic and plasmid DNA were isolated from four different JC1 lysogen isolates and wildtype Van1 and analyzed on an agarose gel. No significant differences were observed between the strains on the gels, suggesting JC1 likely is not taking the form of a phagemid (data not shown).

**Figure 7:**
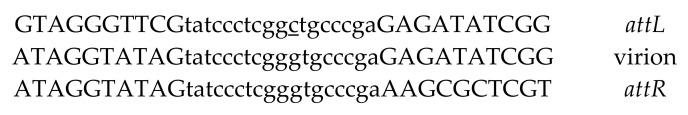
Sequence of JC1 attP overlap region in Burkholderia cenocepacia strain Van1. The 18 bp overlapping sequence present in attL and attR of the JC1 prophage and in the chromosome of the phage (virion) is lowercase. JC1 attP site is located 41 bp upstream of gp1, a predicted serine recombinase. The 1 bp difference between the attB site is underlined.

To identify the Van1 genome location where JC1 is integrating, we used a protocol from Williams et al. [56]. We determined that JC1 integrates into the 5′ end of the conserved gene *rimO* using an 18 bp *attP* overlapping region with 1 bp difference in the *attB* site (Figure 7). RimO belongs to the methylthiotransferase (MTTase) family of proteins and is involved in β-methylthiolation of residue D88 of the ribosomal S12 protein [99]. To further support these findings, the prophage found in the four *B. multivorans* genomes discussed in the genomic characterization section is also located directly next to *rimO,* with part of its integrase gene overlapping with *rimO*. Since JC1 integration disrupts the sequence of *rimO* so early into the coding region, loss of function seemed likely. However, with closer examination of the region an ATG is found 15 bp upstream of the *attP* site in the JC1 genome that allows RimO in Van1 to remain in frame while only changing 6 of the first 10 amino acids, and no change to the overall length of the protein (Supplementary Figure S1). It is possible that the amino acid changes or the phage DNA upstream of the start site could affect the expression or function of RimO, but highly conserved amino acids identified in the N-terminal region among the MTTase family are not affected by JC1 integration [99], and we predict RimO likely remains functional.

Previous studies have found increased resistance when residues around D88 of the S12 ribosomal protein are mutated [100], but similar to previous studies examining *rimO* knockout mutants [99,101], we saw no difference in streptomycin resistance between the JC1 lysogen and wildtype Van1 (data not shown). Since we do not know if or how activity of RimO is affected by JC1 integration, this data only confirms that resistance to streptomycin is not altered by JC1 integration. This is the first account of stable integration identified for the Bcep22-like phages, and it is possible that other members of this phage group may integrate next to *rimO* in an appropriate host. As briefly noted above, Bcep22, BcepIL02, and DC1 have not had successful attempts to isolate stable lysogens in host strains *B. cenocepacia* PC184 and AU1054 [62,63]. This could be due to several reasons, including a non-functional recombinase or bacterial strain incompatibility. Additionally, none of the other phages harbour the *attP* site that JC1 contains, and therefore their *attP* sites and integration locations may be different (or non-existent) than that of JC1.

### 3.10. Virulence Index of JC1

**Figure 8:**
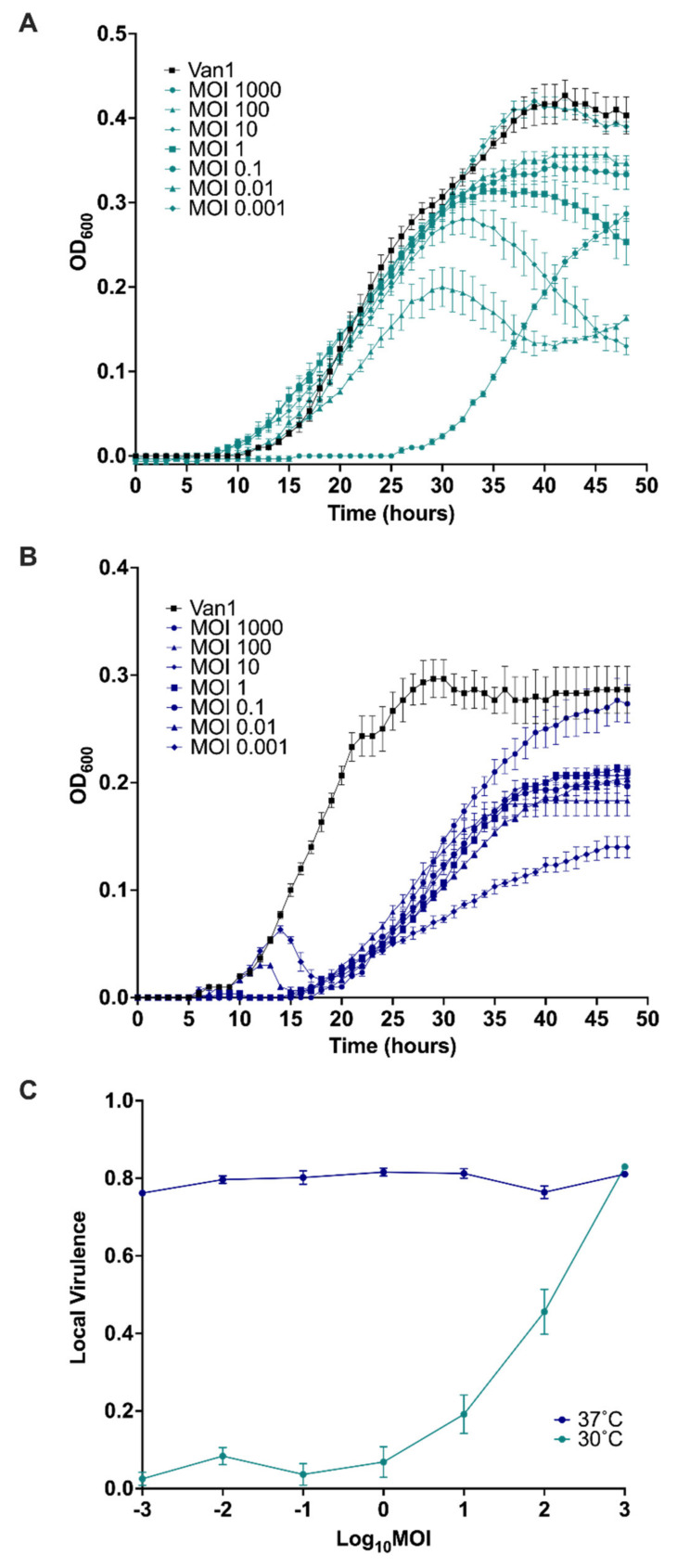
Virulence of JC1 against *Burkholderia cenocepacia* Van1 at (**A**) 30 °C versus (**B**) 37 °C. Kill curves were measured every hour for 48 h. (**C**) Virulence curves of JC1 at 30 °C and 37 °C were calculated by dividing the area under the curve for each MOI by the area under the curve of the bacterial control and subtracting that from 1. A virulence index of 0 to 1 signifies a complete absence of virulence to a theoretical maximum virulence, respectively. All error bars represent the standard error of the mean (SEM). Data from three biological replicates is shown.

In addition to the qualitative observations done in this study we decided to provide a quantitative measure of JC1 virulence against its host strain. As mentioned by Storms et al. [102], characterizing novel phage tends to focus on non-standardized methods when looking at phage virulence. Troubleshooting in the lab to get high titer JC1 stocks (~10^10^ PFU/mL) led us to discover JC1 propagates to a higher titer at 30 °C as opposed to 37 °C; an interesting discovery given the delay it takes for JC1 to form plaques at 30 °C as mentioned above. This discrepancy led us to look at the virulence index for JC1 at 30 °C and 37 °C (Figure 8). The most effective MOI at 30 °C was 1000, though a significant amount of outgrowth occurred at this MOI, leaving MOIs 100 and 10 being the most effective at the 48-h endpoint (Figure 8A). As expected, the least effective MOI at 30 °C was 0.001, almost matching the growth of the bacterial control. Surprisingly, the least effective MOI at 30 °C (MOI 0.001) reduces the most growth by the 48-h endpoint at 37 °C and the highest MOI of 1000 had the most outgrowth, almost reaching bacterial control levels at 48 h (Figure 8B).

Using the equations from Storms et al. [102], the area under the curve for each MOI was calculated from time point 0 to the onset of stationary phase in the bacterial control. Time points 0–40 h and 0–30 h were used to calculate the local virulence for each MOI (log MOI −3 to 3) at 30 °C and 37 °C, respectively. JC1’s local virulence at each MOI for both temperatures were plotted (Figure 8C) and the global virulence index is 0.21 and 0.8 at 30 °C and 37 °C, respectively. JC1 activity against Van1 seems mostly unaffected by MOI at 37 °C, maintaining a virulence above 0.75 for every MOI tested. JC1’s global virulence index at 37 °C is more comparable to lytic *E. coli* phage T7 (0.84) than to lysogenic *E. coli* phage T5 (0.17) [102]. It is important to note that a different range of MOIs were used to test these phages (log MOI −7 to 0), and comparisons can only be made at similar MOIs. With that said, local virulence for T5 does not begin to match the level of virulence seen with JC1 at 37 °C until an MOI of 1 [102]. These similarities are the opposite when we look at virulence for 30 °C, where the curve and global virulence index is less virulent than T5 at comparable MOIs [102]. These results suggest that JC1 could be acting more lytic at 37 °C and more lysogenic at 30 °C. Furthermore, these results explain why JC1 reaches a higher titer when propagated at 30 °C, as a lower virulence is useful when propagating phage to high titer because the bacterial population is not reduced completely, and the phage have sufficient host cells to propagate on.

The cause of this surprising discrepancy in virulence at different temperatures is unknown but could potentially be due to a temperature sensitive switch between lytic and lysogenic lifestyle similar to podophage ØBp-AMP1 [103]. However, no bacterial lysis is seen when overlays of the JC1 lysogen are incubated at 30 °C or 37 °C (data not shown), suggesting a temperature switch may not be the cause of this discrepancy in virulence. Furthermore, infection efficiency and lysogen stability are not affected by a change in temperature (data not shown), though this does not rule out the chance JC1 lysogenizes at a higher rate at 30 °C. It is also possible that the difference in virulence is an effect of the bacterial growth rate, as Van1 grows slower but to an overall higher density at 30 °C, and grows faster but to an overall lower density at 37 °C. Further experiments are required to determine the cause of this shift in virulence seen at 30 °C and 37 °C.

### 3.11. Lysogenic Conversion

One of the main reasons lytic phages are favoured for therapy is they cannot alter bacterial virulence with phage gene expression [104,105]. *Burkholderia* phages tend not to encode recognizable toxins or virulence factors but are known to encode proteins that can contribute to overall fitness [106]. Given the lack of obvious toxins/virulence factors, the significant number of hypothetical gene products with no predicted function, and the presence of moron genes involved in nutrient acquisition like *csrA*, an N-actyltransferase, and a PAPS reductase, we hypothesized JC1 may offer its host cell a growth advantage.

**Figure 9:**
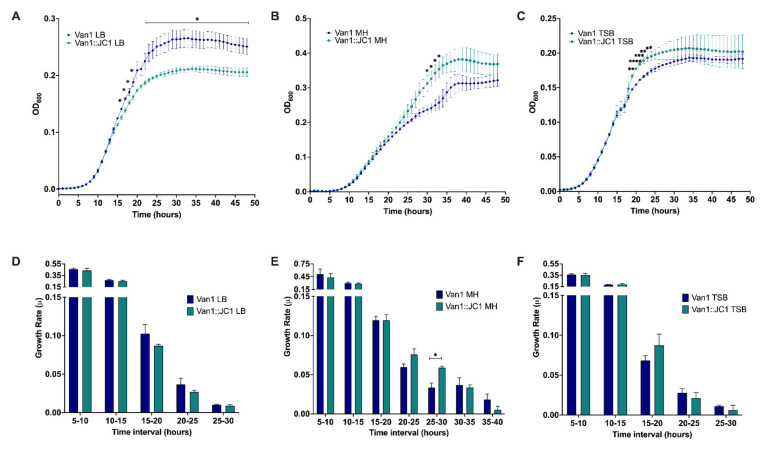
Growth comparison of Van1 versus Van1::JC1 lysogen. (**A**–**C**) Growth curve analysis of wildtype Van1 and Van1::JC1 lysogen in either Luria Bertani (LB), Muller Hinton (MH), or tryptic soy broth (TSB) liquid media. Overnight cultures were subcultured 1:100 in LB for 2 h and 45 min and further diluted 1:100 to a CFU/mL of approximately 1 × 10^6^ in the desired medium and measured every hour for 48 h. (**D**–**F**) Growth rate of curves in (**A**–**C**) calculated using growth rate equation log_10_N − log_10_N_0_ = (μ/2.303) (t − t_0_). Statistical analysis was performed using unpaired *t*-tests (* *p* < 0.05; ** *p* < 0.01; *** *p* < 0.001; **** *p* < 0.0001). All error bars represent the standard error of the mean (SEM). Data from three biological replicates is shown.

Examining the growth of the lysogen verses wildtype Van1 in three different rich mediums showed a difference in growth between the two strains (Figure 9A–C). The lysogen exhibited moderately increased growth after 48 h in MH and TSB liquid medias while exhibiting a slight decrease in growth in LB liquid media. A statistically significant difference in growth was observed between the lysogen and wildtype Van1 at 15–18 and 23–48 h when grown in LB (*p* < 0.05), at 30–33 h when grown in MH (*p* < 0.05), and at 19–24 h when grown in TSB (*p* < 0.05; *p* < 0.01; *p* < 0.001; *p* < 0.0001). To further examine the difference in growth we calculated the growth rate during log phase for each strain in each medium (Figure 9D–F); since the bacteria reach stationary phase at different time points in each media, the time points analyzed vary. A statistically significant difference in growth rate is observed between 25 and 30 h in MH (*p* < 0.05), further supporting the increase in growth exhibited by the lysogen at this time interval (Figure 9B,E). Overall, growth rate is not substantially affected in LB or TSB media, which is clear in the similar shapes of the curves for each strain in each media (Figure 9A,C,D,F). The largest difference in growth in LB and TSB begins when the bacterial growth is beginning to slow, resulting in lower growth rates that are closer together, even though the actual bacterial density is significantly different from one another (Figure 9A,C,D,F).

Lysogenic conversion is not well studied in *Burkholderia* species, and when done so typically examines change in virulence [78,104]. Two significant reasons for the paucity of studies in this area are that many of the characterized *Burkholderia* phages have been isolated from lysogens and therefore lack a wildtype strain for comparison [40,74,80,106,107,108,109], and/or attempts to create stable lysogens in other strains were unsuccessful [62,63]. A third reason is that most phage characterization studies are done with intent of using the phages for therapy and therefore favour the idea of removing lysogeny genes as opposed to studying lysogenic conversion [80,104]. To our knowledge, growth rate differences have not been studied in any *Burkholderia* lysogens but have been observed in *Stenotrophomonas maltophilia* lysogenized by bacteriophage DLP3 [58] and by ϕ24B integration in *E. coli* B strain MC1061 [110]. The differences observed between the growth of the Van1::JC1 lysogen and wildtype Van1 in each media demonstrates potential differences in energy utilization or nutrient acquisition.

### 3.12. Comparison of Van1 & Van1::JC1 in Galleria mellonella and Normal Human Serum (NHS)

**Figure 10:**
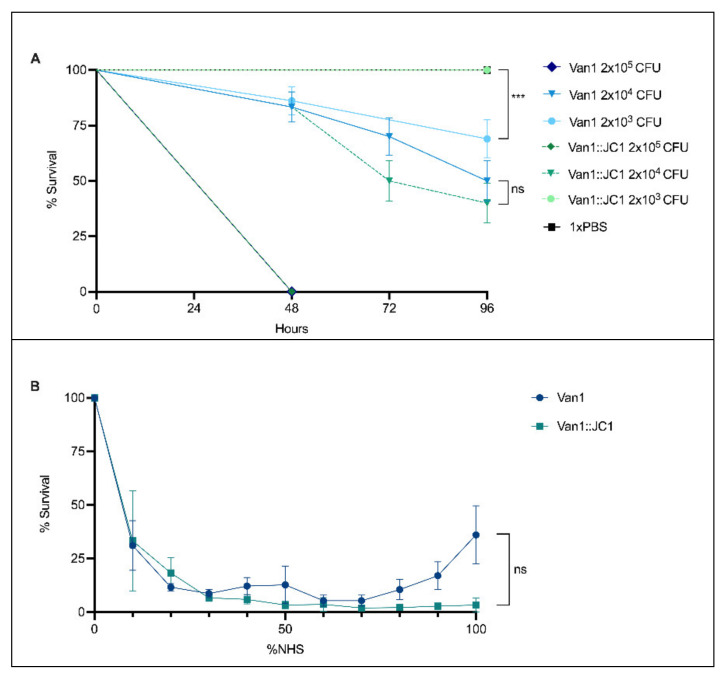
Comparison of Van1 and Van1::JC1 lysogen in *Galleria mellonella* and Normal human serum (NHS). (**A**) Percent survival of *G. mellonella* over 96 h following infection with Van1 or Van1::JC1 lysogen at varying CFU. Larvae of similar size were injected with 5 μL of desired bacterial strain or 1xPBS, incubated at 37 °C, and monitored every 24 h for survival. Statistical analysis was performed using Logrank (Mantel-Cox test) (ns; no significance, ***; *p* < 0.001). Error bars represent standard error. Data from 3 biological replicates is shown, with ten larvae injected per replicate. (**B**) Percent survival of Van1 and Van1::JC1 lysogen in various concentrations of NHS. Bacteria were inoculated (10^4^–10^5^ CFU) into various concentrations of NHS diluted with LB. CFU counts were collected after a 2h incubation at 37 °C with humidity. Statistical analysis was performed using unpaired t-tests (ns; no significance). Error bars represent standard error of the mean (SEM). Data from three biological replicates is shown.

The differences between the Van1::JC1 lysogen and the wildtype strain in different media led us to examine whether changes in growth could have an effect on the virulence of Van1 *in vivo*. To study whether a difference in virulence exists between *B. cenocepacia* Van1 and the Van1::JC1 lysogen we injected the larval infection model *G. mellonella* [111,112,113] with various CFUs of each strain (Figure 10A). Van1 wildtype and the JC1 lysogen show dose-dependent killing, with a lethal dose of 2 × 10^3^ CFU and all wax worms dead by 48 h. The LD_50_ (50% lethal dose) is 2 × 10^4^ CFU, with no statistically significant difference seen between both strains. Interestingly, at 2 × 10^5^ CFU the Van1::JC1 lysogen is completely avirulent, whereas the wildtype strain is still able to kill about 25% of the population (*p* < 0.001). Given that we saw both increased and decreased growth depending upon which media the lysogen was grown in (Figure 9), it appears that the Van1::JC1 lysogen could be experiencing a slight growth disadvantage in the wax worm infection model, observable only at low CFU. Alternatively, the larval immune response could be causing stress on the lysogen at a low CFU resulting in a prophage switch to the lytic cycle, and therefore fewer viable Van1::JC1 bacteria. Further experiments are required to fully understand the lack of virulence in wax worms observed at the lower CFU for Van1::JC1.

“Cepacia Syndrome”, characterized by necrotizing pneumonia and septicaemia, is most commonly associated with *B. cenocepacia* infections [3,114,115]. To further explore the difference in fitness exhibited by wildtype Van1 and the Van1::JC1 lysogen, we tested the ability of each strain to survive in different concentrations of NHS (Figure 10B). NHS contains the heat-labile principle known as the complement system, a significant component of our innate immune system [116]. Overall, both strains do not show high propensity for survival in NHS, but wildtype Van1 demonstrates a higher percent survival between 80–100% NHS than the lysogen (*p* > 0.05; no significance). Given the lack of significance, no conclusions can be drawn, but there does seem to be a slight disadvantage for the lysogen in NHS, which coincides with our findings of decreased virulence in the *G. mellonella* infection model. This minor decrease in survival when exposed to complement could in part explain the decrease in virulence observed for the Van1::JC1 lysogen. The innate immune response of *G. mellonella* has complement-like proteins, and virulence studies from *G. mellonella* show strong correlation to results obtained from mice and rat lung [111,117,118,119]. Potentially, prophage JC1 may increase the sensitivity of Van1 towards the innate immune response, however, further experiments are required before solid conclusions can be drawn about this relationship.

## 4. Conclusions

Aside from encoding a repressor and an integrase, and being able to stably lysogenize its host bacterium, phage JC1 possesses several characteristics that would make it ideal for use in phage therapy. For one, JC1 has a considerably broad host range, being able to infect many member species of the Bcc. Phage JC1 also requires the inner core of the LPS for infection, so bacteria that mutate to evade infection by eliminating their O-antigen will likely develop a fitness disadvantage, as observed in previously characterized LPS mutants that lack the inner core [66,67]. Another favorable factor is the high level of virulence JC1 exhibits against host strain Van1 at 37 °C, which is comparable to virulence exhibited by lytic *E. coli* phage T7 at similar MOIs [102]. If the decrease in virulence at 30 °C is caused by an increased rate of lysogeny, then deletion of the genes responsible for lysogeny should increase the virulence index of JC1 at 30 °C and would make JC1 more suitable for use in therapy. Further experiments examining these potential alterations to make JC1 a suitable candidate for phage therapy against the Bcc are currently being conducted.

## Figures and Tables

**Table 1:** Host range analysis of JC1 on 85 Burkholderia strains.

Burkholderia Species	Strain	Efficiency of Plating (EOP)	Source/Reference
*B. cepacia*	ATCC 25416 ^T^	ND	Onion/[13,14,15]
	ATCC 17759	ND	Soil, Trinidad/[13,14,15,16]
	CEP509/LMG 18821	6.7 × 10^−7^	CF patient, Australia/[13]
	CEP521	7.3 × 10^−7^	CF patient, Canada/CBCCRRR *
*B. multivorans*	ATCC 17616	+++	Soil, USA/[13,16,17,18,19]
	C3430	ND	CF patient, Canada/[20]
	C1576, LMG 16660	ND	CF-e patient, UK/[13,19,21]
	C5274	8.0 × 10^−6^	CF patient, Canada/[20]
	C5393	ND	CF patient, Canada/[13,20]
	C5568	++	CF patient, Canada/[20]
	JC1	++	CF patient, Canada/[20]
	LMG 13010 ^T^	++	CF patient, Belgium/[13,19,20,21,22,23]
	M1512	ND	CF patient, Canada/[24]
	M1865	ND	CF patient, Canada/[24]
	R810	ND	CF patient, Canada/[24]
	R1159	ND	CF patient, Canada/[24]
*B. cenocepacia*	AU1054	0.93	CF patient/[18]
	715j	ND	CF patient, USA/[25]
	BS1	++	CF patient, Canada/This study
	BS2	++	CF patient, Canada/This study
	BS3	++	CF patient, Canada/This study
	C1257	++	CF-e patient, USA/[20]
	C4455	++	CF-e patient, Canada/[20]
	C5424	0.25	CF-e patient, Canada/[13,21]
	C6433	ND	CF-e patient, Canada/[13,21,26]
	C8963	0.3	CF patient, Canada/[27]
	C9343	ND	CF patient, Canada/[27]
	CEP511	++	CF-e patient, Australia/[13,22]
	CEP0868	0.002	CF patient, Argentina/[25]
	D1	ND	Soil, USA/[24]
	HI2424	ND	Soil, USA/[24]
	J2315	0.14	CF-e patient, UK/[13,28,29]
	K56-2	+++	CF-e patient, Canada/[13,30]
	K63-3	ND	CF-e patient, Canada/[30]
	LMG 19240	ND	Wheat soil, Australia/[31]
	MCO-3	ND	Maize soil, USA/[32]
	PC184	0.19	CF-e patient, USA/[13,33]
	R161	+	CF patient, Canada/[24]
	R452	+	CF patient, Canada/[24]
	R750	0.9	CF patient, Canada/[24]
	R1284	0.022	CF patient, Canada/[24]
	R1285	0.6	CF patient, Canada/[24]
	R1314	++	CF patient, Canada/[24]
	R1434	0.86	CF patient, Canada/[24]
	R1619	++	CF patient, Canada/[24]
	R1882	0.53	CF patient, Canada/[24]
	R1883	0.47	CF patient, Canada/[24]
	R1884	1	CF patient, Canada/[24]
	R2314	0.73	CF patient, Canada/[24]
	RK1b	0.31	CF patient, Canada/[24]
	S11528	0.8	CF patient, Canada/[24]
	Van1	1	CF patient, Canada/This study
*B. stabilis*	LMG 14294	+	CF patient, Belgium/[13,23]
	C7322/LMG 18870	6.0 × 10^−4^	CF patient, Canada/[13,22]
	R450	ND	CF patient, Canada/[24]
	R2140	+	CF patient, Canada/[24]
	R2339	ND	CF patient, Canada/[24]
*B. vietnamiensis*	DBO1	ND	Soil, USA/[34]
	LMG 10929 ^T^	ND	Rice, Vietnam/[13,19,35]
	PC259/LMG 18835	+	CF patient, USA/[13,36,37]
	G4	ND	Soil, USA/[38]
*B. dolosa*	AU0158	++	CF patient, USA/[39]
	CEP021	+	CF patient, USA/[39]
	E12	8.6 × 10^−5^	CF patient, UK/[39]
	STM1441	ND	Soil, Senegal/[39]
*B. ambifaria*	AMMD ^T^	ND	Soil, USA/[39]
	ATCC 53266	2.3 × 10^−5^	Soil, USA/[39]
	CEP996	0.31	CF patient, Australia/[39]
	M53	ND	Soil, USA/[24]
*B. anthina*	AU1293	0.8	CF patient, USA/[39]
	C1765	4.3 × 10^−3^	CF patient, UK/[39]
	J2552	ND	Soil, UK/[39]
	W92^T^	ND	Soil, USA/[39]
*B. pyrrocinia*	ATCC 15958	ND	Soil, Japan/[39]
	ATCC 39277	ND	Soil, USA/[39]
	BC011	ND	Water, USA/[39]
	C1469	ND	CF patient, UK/[39]
Bcc Group K	CEP0964	ND	CF patient, Canada/[24]
	CEP1056	++	CF patient, Canada/[24]
	R445	3.1 × 10^−5^	CF patient, Canada/[24]
*B. lata*	383	ND	Soil, Trinidad/[40]
*Burkholderia* sp.	JS150	1	Soil, USA/[24]
*Pandoraea* sp.	R1717	ND	CF patient, Canada/[24]
Ralstonia pickettii	ATCC 27511	0.7	Patient isolate, USA/[41]
	YH105	ND	Soil, USA/[42]

ND, Not detected; +, clearing at 10^10^ PFU/mL; ++, clearing at 10^9^ PFU/mL; +++, clearing at 10^8^ PFU/mL. EOP calculated by dividing PFU/mL on each strain by actual PFU/mL determined on strain Van1. Abbreviations: ^T^, type strain; CF, cystic fibrosis isolate; CF-e, cystic fibrosis epidemic isolate. * Canadian Burkholderia cepacia complex Research and Referral Repository.

**Table 2:** JC1 receptor identification on *B. cenocepacia* K56-2 LPS mutants.

Bacterial Strain	Phenotype	pSCRhaB2	pSCRhaB2-Complement
K56-2	Wildtype LPS	+	+
SAL1	K56-2 *hldA*:: pSL5, Lacks inner and outer core	–	+
CCB1	K56-2 *waaC*::pGPΩTp, Lacks inner and outer core	–	+
XOA8	K56-2 *wabO*::pGPΩTp, Lacks inner and outer core	–	+
XOA7	K56-2 *waaL*::pGPΩTp, Lacks outer core	+	+
XOA15	K56-2 *wabR*::pGPΩTp, Lacks outer core	+	+
XOA17	K56-2 *wabS*::pGPΩTp, Lacks outer core	+	+
RSF19	K56-2 *wbxE*:: pRF201, Lacks O-antigen	+	+

**Table 3:** Bacteriophage genome annotations for JC1 obtained from BLASTp data.

Gene	Start	End	Strand	Length(aa)	Putative Function	BLASTp Hit	Species	Coverage (%)	E-Value	Identity (%)	Accession
1	1660	5	–	551 aa	serine recombinase	serine recombinase-like protein	*Burkholderia* phage Bcep22	100	0	70.29	NP_944235.2
2	2081	1794	–	95 aa	hypothetical protein	hypothetical protein Bcep22_gp07	*Burkholderia* phage Bcep22	72	2 × 10^−27^	65.22	YP_009173769.1
3	2493	2146	–	115 aa	hypothetical protein	hypothetical protein Bcep22_gp09	*Burkholderia* phage Bcep22	98	2 × 10^−21^	40.35	NP_944237.1
4	2867	2490	–	125 aa	hypothetical protein	hypothetical protein	*Burkholderia multivorans*	100	3 × 10^−84^	96.80	WP_217093966.1
5	3895	2864	–	343 aa	RecT-like protein	RecT-like protein	*Burkholderia* phage Bcep22	100	1 × 10^−178^	74.16	NP_944238.1
6	4976	3945	–	343 aa	nuclease/RecB-like protein	nuclease/RecB-like protein	*Burkholderia* phage Bcepmigl	98	0	84.32	YP_007236753.1
7	5697	5104	–	197 aa	repressor	transcriptional regulator	*Burkholderia* phage Bcepmigl	100	7 × 10^−81^	59.90	YP_007236754.1
8	5821	6165	+	114 aa	transcriptional regulator	transcriptional regulator	*Burkholderia* phage DC1	78	8 × 10^−38^	66.29	YP_006589939.1
9	6335	6685	+	116 aa	hypothetical protein	hypothetical protein G167_gp75	*Burkholderia* phage Bcepmigl	96	4 × 10^−67^	87.50	YP_007236756.1
10	6737	7546	+	269 aa	virion-associated protein ^a^	hypothetical protein BcepIL02_gp11	*Burkholderia* phage Bcepil02	99	2 × 10^−143^	73.98	YP_002922683.1
11	7642	8142	+	166 aa	single stranded DNA binding protein	single stranded DNA binding protein	*Burkholderia* phage DC1	100	4 × 10^−82^	81.33	YP_006589943.1
12	8151	8369	+	72 aa	hypothetical protein	hypothetical protein B862_gp69	*Burkholderia* phage DC1	100	3 × 10^−40^	88.89	YP_006589944.1
13	8366	8842	+	158 aa	hypothetical protein	hypothetical protein UAM5_00057	*Ralstonia* phage UAM5	99	2 × 10^−68^	65.61	CAH0532174.1
14	8839	9201	+	120 aa	hypothetical protein	hypothetical protein KMC44_gp61	*Ralstonia* phage Cimandef	98	1 × 10^−59^	70.87	YP_010078217.1
15	9352	9807	+	151 aa	hypothetical protein	hypothetical protein B862_gp66	*Burkholderia* phage DC1	84	7 × 10^−68^	85.94	YP_006589947.1
16	10,127	9801	–	108 aa	hypothetical protein	hypothetical protein	*Burkholderia multivorans*	100	2 × 10^−75^	100	WP_217093979.1
17	10,389	10,132	–	85 aa	hypothetical protein	hypothetical protein	*Burkholderia multivorans*	100	2 × 10^−53^	100	WP_217093980.1
18	10,744	10,974	+	76 aa	hypothetical protein	hypothetical protein B862_gp65	*Burkholderia* phage DC1	100	3 × 10^−39^	84.21	YP_006589948.1
19	10,971	11,357	+	128 aa	helicase	TPA: MAG TPA: hypothetical protein	*Siphoviridae* sp.	61	6 × 10^−18^	44.30	DAT31939.1
20	11,354	12,178	+	274 aa	eplication initiator protein	replication protein	*Burkholderia* phage Bcepmigl	39	2 × 10^−48^	76.85	YP_007236768.1
21	12,175	12,975	+	266 aa	DnaC-like protein	DnaC-like protein	*Burkholderia* phage Bcepil02	99	4 × 10^−150^	76.60	YP_002922693.1
22	13,057	13,182	+	41 aa	hypothetical protein	hypothetical protein G167_gp61	*Burkholderia* phage Bcepmigl	100	1 × 10^−9^	56.10	YP_007236770.1
23	13,337	13,765	+	142 aa	hypothetical protein	hypothetical protein B862_gp60	*Burkholderia* phage DC1	98	5 × 10^−51^	60.28	YP_006589953.1
24	13,771	14,106	+	111 aa	hypothetical protein	hypothetical protein BcepIL02_gp24	*Burkholderia* phage Bcepil02	85	1 × 10^−16^	43.75	YP_002922696.1
25	14,166	14,609	+	147 aa	hypothetical protein	TPA: MAG TPA_asm: hypothetical protein	*Myoviridae* sp.	95	7 × 10^−44^	49.29	DAL29776.1
26	14,652	15,041	+	129 aa	hypothetical protein	TPA: MAG TPA: hypothetical protein	*Myoviridae* sp.	95	1 × 10^−9^	34.35	DAP81611.1.1
27	15,044	15,325	+	93 aa	hypothetical protein	hypothetical protein B862_gp58	*Burkholderia* phage DC1	100	3 × 10^−40^	70.83	YP_006589955.1
28	15,364	16,602	+	412 aa	hypothetical protein	hypothetical protein Bcep22_gp31	*Burkholderia* phage Bcep22	100	6 × 10^−156^	62.42	NP_944260.2
29	17,017	16,658	–	119 aa	hypothetical protein	hypothetical protein KMC50_gp40	*Ralstonia* phage Claudette	85	2 × 10^−25^	49.02	YP_010078630.1
30	17,210	18,010	+	266 aa	terminase small subunit	terminase small subunit	*Burkholderia* phage DC1	93	8 × 10^−161^	83.53	YP_006589958.1
31	18,090	18,338	+	82 aa	hypothetical protein	hypothetical protein B862_gp53	*Burkholderia* phage DC1	92	2 × 10^−39^	82.89	YP_006589960.1
32	18,389	18,517	+	42 aa	hypothetical protein	hypothetical protein B862_gp49	*Burkholderia* phage DC1	97	2 × 10^−17^	87.80	YP_006589964.1
33	18,562	18,840	+	92 aa	hypothetical protein	hypothetical protein	*Burkholderia multivorans*	100	1 × 10^−59^	97.83	WP_217093993.1
34	18,872	19,066	+	64 aa	hypothetical protein	hypothetical protein	*Burkholderia multivorans*	100	9 × 10^−36^	96.88	WP_217093994.1
35	19,059	20,366	+	435 aa	hypothetical protein	hypothetical protein phiE131_040	*Burkholderia* phage phiE131	47	9 × 10^−23^	41.40	AYJ74306.1
36	20,356	20,766	+	136 aa	hypothetical protein	hypothetical protein HOT12_gp34	*Burkholderia* phage vB_BmuP_KL4	84	5 × 10^−32^	68.10	YP_009800723.1
37	20,851	21,033	+	60 aa	hypothetical protein	hypothetical protein	*Burkholderia multivorans*	100	8 × 10^−33^	100	WP_217093996.1
38	21,030	21,416	+	128 aa	hypothetical protein	TPA: MAG TPA: Protein of unknown function (DUF2591)	*Caudovirales* sp.	99	2 × 10^−13^	36.76	DAH87964.1
39	21,413	21,700	+	95 aa	hypothetical protein	hypothetical protein	*Burkholderia multivorans*	100	9 × 10^−63^	100	WP_217093998.1
40	21,697	22,035	+	112 aa	hypothetical protein	hypothetical protein Bcep22_gp48	*Burkholderia* phage Bcep22	99	2 × 10^−65^	85.59	NP_944277.1
41	22,172	22,582	+	136 aa	DUF2778 domain-containing protein	TPA: MAG TPA: Protein of unknown function (DUF2778)	*Myoviridae* sp.	98	8 × 10^−19^	39.57	DAO56318.1
42	22,579	22,860	+	93 aa	hypothetical protein	hypothetical protein	*Burkholderia multivorans*	100	7 × 10^−57^	98.92	WP_217094000.1
43	22,948	24,549	+	533 aa	terminase large subunit	terminase large subunit	*Burkholderia* phage DC1	98	0	83.11	YP_006589971.1
44	24,560	24,991	+	143 aa	hypothetical protein	hypothetical protein BcepIL02_gp45	*Burkholderia* phage Bcepil02	99	2 × 10^−82^	82.39	YP_002922717.1
45	25,013	27,310	+	765 aa	portal protein	phage portal protein	*Burkholderia* phage Bcepil02	95	0	77.53	YP_002922718.1
46	27,318	28,340	+	340 aa	virion-associated protein^a^	hypothetical protein Bcep22_gp52	*Burkholderia* phage Bcep22	98	3 × 10^−110^	57.82	NP_944281.1
47	28,367	28,561	+	64 aa	carbon storage regulator	carbon storage regulator	*Burkholderia* phage DC1	100	1 × 10^−30^	89.06	YP_006589976.1
48	28,660	29,754	+	364 aa	major capsid protein	major capsid protein	*Burkholderia* phage Bcepmigl	100	0	92.03	YP_007236797.1
49	29,821	30,288	+	155 aa	virion-associated protein	virion associated protein	*Burkholderia* phage Bcepmigl	100	2 × 10^−77^	74.84	YP_007236798.1
50	30,346	30,942	+	198 aa	hypothetical protein	hypothetical protein B862_gp33	*Burkholderia* phage DC1	99	5 × 10^−80^	63.41	YP_006589980.1
51	30,946	31,593	+	215 aa	virion-associated protein ^a^	hypothetical protein B862_gp32	*Burkholderia* phage DC1	100	7 × 10^−147^	92.09	YP_006589981.1
52	31,590	32,219	+	209 aa	virion-associated protein ^a^	hypothetical protein G167_gp30	*Burkholderia* phage Bcepmigl	100	6 × 10^−117^	77.14	YP_007236801.1
53	32,229	32,648	+	139 aa	virion-associated protein	major capsid protein	*Burkholderia* phage DC1	100	6 × 10^−93^	94.24	YP_006589983.1
54	32,653	33,528	+	291 aa	hypothetical protein	hypothetical protein B862_gp29	*Burkholderia* phage DC1	97	9 × 10^−147^	74.39	YP_006589984.1
55	33,510	33,788	+	92 aa	virion-associated protein	virion-associated phage protein	*Burkholderia* phage Bcepil02	100	2 × 10^−53^	90.22	YP_002922729.1
56	33,790	34,740	+	316 aa	tail fiber protein	putative tail fiber protein	*Burkholderia* phage Bcepil02	100	4 × 10^−177^	76.90	YP_002922730.1
57	34,744	35,814	+	356 aa	tail fiber protein	putative tail fiber protein	*Burkholderia* phage Bcepil02	100	2 × 10^−145^	64.54	YP_002922731.1
58	35,811	36,323	+	170 aa	hypothetical protein	hypothetical protein BcepIL02_gp60	*Burkholderia* phage Bcepil02	98	7 × 10^−39^	45.29	YP_002922732.1
59	36,484	37,530	+	348 aa	tail fiber protein	TPA: MAG TPA: Endo N acetylneuraminidase	*Siphoviridae* sp.	59	9 × 10^−84^	63.59	DAM52127.1
60	37,532	38,221	+	229 aa	hypothetical protein	hypothetical protein	*Pseudomonas* phage Dolphis	100	6 × 10^−18^	44.92	QNJ57341.1
61	38,276	40,018	+	580 aa	head closure protein	virion-associated phage protein	Burkholderia phage Bcepil02	100	0	90.00	YP_002922735.1
62	40,020	40,373	+	117 aa	hypothetical protein ^a^	virion-associated phage protein	*Burkholderia* phage Bcepil02	97	2 × 10^−62^	86.09	YP_002922736.1
63	40,424	40,864	+	146 aa	acetyltransferase	acetyltransferase	*Burkholderia* phage DC1	97	6 × 10^−92^	89.51	YP_006589993.1
64	40,857	41,855	+	332 aa	virion-associated protein ^a^	hypothetical protein B862_gp19	*Burkholderia* phage DC1	100	0	91.27	YP_006589994.1
65	41,867	42,589	+	240 aa	phosphoadenosine phosphosulfate reductase	phosphoadenosine phosphosulfate reductase	*Burkholderia* phage DC1	100	2 × 10^−167^	94.17	YP_006589995.1
66	42,589	42,888	+	99 aa	hypothetical protein	hypothetical protein	*Burkholderia multivorans*	100	2 × 10^−59^	100	WP_217094022.1
67	42,905	43,495	+	196 aa	virion-associated protein	virion-associated phage protein	*Burkholderia* phage Bcepil02	30	1 × 10^−4^	49.21	YP_002922740.1
68	43,506	45,038	+	510 aa	virion-associated protein ^a^	hypothetical protein B862_gp17	*Burkholderia* phage DC1	100	0	80.30	YP_006589996.1
69	45,123	58,670	+	4515 aa	DarB-like antirestriction protein	DarB-like antirestriction protein	*Burkholderia* phage Bcep22	100	0	79.33	NP_944303.1
70	58,940	58,701	–	79 aa	hypothetical protein	hypothetical protein BcepIL02_gp71	*Burkholderia* phage Bcepil02	97	1 × 10^−46^	89.61	YP_002922743.1
71	59,287	58,991	–	98 aa	hypothetical protein	hypothetical protein G167_gp14	*Burkholderia* phage Bcepmigl	100	2 × 10^−41^	67.35	YP_007236817.1
72	59,443	59,790	+	115 aa	LydA-like holin	LydA-like holin	uncultured *Caudovirales* phage	82	4 × 10^−27^	54.74	CAB4121548.1
73	59,787	60,059	+	90 aa	holin	holin	*Burkholderia* phage vB_BceS_AH2	91	6 × 10^−32^	68.29	YP_006561127.1
74	60,056	60,634	+	192 aa	lysozyme	hypothetical protein AXJ08_gp22	*Rhodoferax* phage P26218	94	7 × 10^−57^	49.45	YP_009222572.1
75	60,631	61,140	+	169 aa	Rz	Rz-like phage lysis protein	*Burkholderia* phage Bcep22	95	7 × 10^−65^	66.27	NP_944308.1
76	60,866	61,087	+	73 aa	Rz1	Rz1	*Burkholderia* phage DC1	100	9 × 10^−16^	83.56	YP_006590003.1

^a^ Putative function determined by mass spectrometry analysis.

**Table 4:** Predicted Rho-independent terminators in JC1.

Start	Program	Strand	Sequence	−ΔG
1753	Both	–	ATCGACTCCAACGGCACCCTCGCGGTGCCGTTTTTATTGCCC	−13.20
6258	Rnamotif	+	CCAGCTGTTGAGCCTCCCGTTTCAGGGAGGCTTTTTGCCCGTA	−15.70
10,407	Rnamotif	–	AGAGCGTCGTCGGCGGCCCGCACGGCCGCCaTTTTTTTCGATC	−16.00
13,228	Rnamotif	+	GGCGACTTTGGTGGGCGGCTCGTACAGCGCCCGTTTTTTTTCACC	−9.60
13,893	Rnamotif	–	CCGATGCGCACCGGCCGGATGTGGCTGATCCGGTTGTTGTATTCGCGG	−10.50
47,347	Rnamotif	–	TCGGCCGACACCTTGCGGCGCTCGGCCGTGAGcaTCTTGTTCCAGC	−12.10
51,986	Rnamotif	–	CCTCCTGAATCGCGCGCCAGATGGCGCGCTTCTGGTTCGGG	−15.60
61,154	Both	+	GGCTGAGACTTCCCCGGCGCGAGCCGGGGTTTTTTATGCCG	−16.40

Rho-independent terminators were identified using the ARNold [69,70,71,72] program and putative terminators with a ΔG value of −9 kcal/mol or less were retained. DNA predicted to form the loop in the RNA is in red, and DNA predicted to encode an RNA stem is blue.

**Table 5:** The conserved domains found in the 76 gene products of JC1.

Gp	Hit Type	PSSM-ID	Interval	E-Value	Accession	Short Name	Superfamily
1	specific	238206	9–159	1.68 × 10^−24^	cd00338	Ser_Recombinase	cl02788
5	superfamily	413988	31–245	2.47 × 10^−48^	cl04285	RecT superfamily	-
6	superfamily	415607	14–168	6.92 × 10^−17^	cl09232	YqaJ superfamily	-
7	specific	238045	11–63	1.31 × 10^−5^	cd00093	HTH_XRE	cl22854
10	superfamily	413281	20–268	2.41 × 10^−72^	cl02338	DUF2303 superfamily	-
14	superfamily	377777	34–98	6.08 × 10^−8^	cl06229	DUF1364 superfamily	-
18	specific	404897	5–74	3.76 × 10^−19^	pfam14090	HTH_39	cl16606
19	specific	214947	15–88	3.71 × 10^−19^	smart00974	T5orf172	cl15257
20	superfamily	237940	106–141	2.12 × 10^−3^	cl36477	PRK15313 superfamily	-
21	superfamily	422963	72–263	1.95 × 10^−39^	cl38936	P-loop_NTPase superfamily	-
30	specific	397583	19–236	7.93 × 10^−24^	pfam03592	Terminase_2	cl01513
38	superfamily	416328	2–120	1.32 × 10^−21^	cl11584	DUF2591 superfamily	-
40	superfamily	404162	3–86	7.90 × 10^−21^	cl16173	DUF4031 superfamily	-
41	specific	402478	1–125	6.70 × 10^−48^	pfam10908	DUF2778	cl12489
43	superfamily	222858	51–252	3.53 × 10^−09^	cl28557	17 superfamily	-
45	superfamily	293119	57–616	9.79 × 10^−10^	cl24922	P22_portal superfamily	-
45	superfamily	135173	668–765	8.02 × 10^−5^	cl31366	PRK04654 superfamily	-
47	specific	396934	1–44	7.10 × 10^−8^	pfam02599	CsrA	cl00670
48	specific	404189	39–358	8.21 × 10^−100^	pfam13252	DUF4043	cl22542
53	superfamily	412204	29–130	4.16 × 10^−3^	cl00184	CAS_like superfamily	-
57	specific	404724	255–306	3.00 × 10^−13^	pfam13884	Peptidase_S74	cl16452
59	specific	404724	248–304	2.58 × 10^−12^	pfam13884	Peptidase_S74	cl16452
63	specific	224584	9–134	6.86 × 10^−4^	COG1670	RimL	cl34333
65	specific	238846	10–181	5.36 × 10^−24^	cd01713	PAPS_reductase	cl00292
68	superfamily	180240	299–400	9.02 × 10^−7^	cl32090	PRK05759 superfamily	-
69	superfamily	226993	1810–2677	6.59 × 10^−99^	cl18793	COG4646 superfamily	
69	specific	408627	4195–4401	1.56 × 10^−36^	pfam18857	LPD38	cl40138
69	specific	408569	3170–3270	9.95 × 10^−19^	pfam18798	LPD3	cl40093
69	specific	381594	80–192	7.10 × 10^−14^	cd00254	LT-like	cl00222
69	specific	223897	1537–1783	3.77 × 10^−11^	COG0827	YtxK	cl28092
69	superfamily	237171	1358–1500	3.31 × 10^−7^	cl36163	PRK12678 superfamily	-
69	superfamily	223627	2412–2868	2.48 × 10^−5^	cl33945	HepA superfamily	-
69	superfamily	235334	1071–1287	1.59 × 10^−3^	cl35279	PRK05035 superfamily	-
72	specific	406481	24–103	3.77 × 10^−16^	pfam16083	Phage_holin_3_3	cl24062
74	superfamily	226439	2–186	2.17 × 10^−28^	cl34694	ZliS superfamily	-
75	superfamily	419854	54–163	2.39 × 10^−10^	cl22701	Phage_lysis superfamily	-

**Table 6:** Proteins determined to be virion-associated by proteomic analysis of CsCl-purified JC1 virions.

Protein	Score	Coverage	Unique Peptides (#)	Putative Function
gp48	298.44	56.04	27	Major capsid protein
gp69	88.92	15.61	51	DarB-like antirestriction protein
gp49	82.92	50.32	6	Virion-associated protein
gp64	45.94	45.18	9	Hypothetical protein
gp53	30.63	58.99	5	Virion-associated protein
gp68	27.46	17.06	9	Hypothetical Protein
gp45	25.85	22.22	14	Portal protein
gp56	22.88	41.14	7	Tail fiber protein
gp52	19.47	46.41	8	Hypothetical protein
gp61	16.01	11.21	6	Virion-associated phage protein
gp10	13.4	21.56	5	Hypothetical protein
gp55	13.16	48.91	4	Virion-associated phage protein
gp51	12.97	25.58	5	Hypothetical protein
gp67	9.35	42.86	7	Virion-associated phage protein
gp46	4.14	13.82	4	Hypothetical protein
gp47	2.61	18.75	1	Carbon storage regulator

## Data Availability

Not applicable.

## Supplementary Materials

The following supporting information can be downloaded at: https://www.mdpi.com/article/10.3390/v14050938/s1, Table S1: Plasmids used in this study. Table S2: Primers used in this study. Figure S1: RimO protein sequence of Van1 versus JC1 lysogen.
