# Supplementary material for: The Isolation and Characterization of a Broad Host Range Bcep22-like Podovirus JC1
Source: Viruses. 2022 Apr 29;14(5):938. doi: 10.3390/v14050938 (PMC9144972; doi:10.3390/v14050938)
Supplement: Supplementary file 1 [file viruses-14-00938-s001.zip › viruses-1688491-supplementary.pdf]

Supplementary Data

Supplementary Table S1: Plasmids used in this study.

Plasmids	Description	Source
pSCRhaB2-Tc	*Burkholderia cenocepacia* rhamnose-inducible expression vector, Tc^R	(Juárez-Lara, unpublished)
pK56-2*hldAhldD*	pSCRha carrying K56-2 *hldA* and *hldD,* Tc^R	This study
pK56-2*waaC*	pSCRha carrying K56-2 *waaC,* Tc^R	This study
pK56-2*waaL*	pSCRha carrying K56-2 *waaL,* Tc^R	This study
pK56-2*wabO*	pSCRha carrying K56-2 *wabO,* Tc^R	This study
pK56-2*wabR*	pSCRha carrying K56-2 *wabR,* Tc^R	This study
pK56-2*wabS*	pSCRha carrying K56-2 *wabS,* Tc^R	This study
pK56-2*wbxE*	pSCRha carrying K56-2 *wbxE,* Tc^R	This study

Supplementary Table S2: Primers used in this study.

Primer name	Sequence (5'-3')	Function
2F	CTGCTTCTTCGATAGTGGTG	Anneals at 14,813 bp to 14,832 bp of JC1 genome, used to detect presence of JC1 genome in bacteria survivors of JC1 infection
2R	TCGGATTCCTCCTTCTCG	Anneals at 15,729 bp to 15,746 bp of JC1 genome, used to detect presence of JC1 genome in bacteria survivors of JC1 infection
attp_F	TCACGAGCAGGCTATACACG	Anneals at 1237 bp to 1256 bp. Flanks the predicted *attP* site upstream of gp1 serine recombinase.
attp_R	TGCAGCGTACAGACAGTTCC	Anneals at 1850 bp to 1869 bp. Flanks the predicted *attP* site upstream of gp1 serine recombinase.
rimO_F	ATCCCCCAAAGTAGGGTTCG	Anneals at 9 bp to 28 bp of Van1 *rimO gene*. Used to confirm integration site of JC1 with attP_R primer.
rimO_R	CACGGCCTGCATCACTTC	Anneals at 9 bp to 28 bp of Van1 *rimO gene*. Used to confirm integration site of JC1 with attP_F primer.
XOA8_kpnI_F	TAATGGTACCGAACAAAACGGCAAGAATCG	Anneals upstream of K56-2 *wabO* gene. KpnI site underlined.
XOA8_xbaI_R	TTTATCTAGAACCGTCATCTGGAAAGCTG	Anneals downstream of K56-2 *wabO* gene. XbaI site underlined.
CCB1_ kpnI_F	TTTTGGTACCGCCGGGTTTATCTTGAAAAG	Anneals upstream of K56-2 *waaC* gene. KpnI site underlined
CCB1_ xbaI_R	TTTTTCTAGAGACGGGACTTCGATGATCTG	Anneals downstream of K56-2 *waaC* gene. XbaI site underlined.
SAL1_ kpnI_F	TTATGGTACCGATGCACTCGTGATCGTGAC	Anneals upstream of K56-2 *hldA* gene. KpnI site underlined
SAL1_ xbaI_R	TTATTCTAGACTGGATCTCCGAAGAAAACG	Anneals downstream of K56-2 *hldD* gene. XbaI site underlined.
kpnI_F_XOA7	TAATGGTACCTACGTGGCGCACTGAAACAC	Anneals upstream of K56-2 *waaL* gene. KpnI site underlined
xbaI_R_XOA7	TAAATCTAGACGATATGGAACAGCAATCGC	Anneals downstream of K56-2 *waaL* gene. XbaI site underlined.
kpnI_F_RSF19	TAAAGGTACCACAGGTTGTATCGGCGTCTC	Anneals upstream of K56-2 *wbxE* gene. KpnI site underlined
xbaI_R_RSF19	TAAATCTAGAACTGCGCCTGGTTGTAACAC	Anneals downstream of K56-2 *wbxE* gene. XbaI site underlined.
kpnI_F_XOA15	TAAAGGTACCCGATTTCGCTAAAATGGCCC	Anneals upstream of K56-2 *wabR* gene. KpnI site underlined
xbaI_R_XOA15	TAAATCTAGAAGACGGTGTACTACCGCTTC	Anneals downstream of K56-2 *wabR* gene. XbaI site underlined.
kpnI_F_XOA17	TAAAGGTACCATCGGATTCAGTTCCAGCAG	Anneals upstream of K56-2 *wabS* gene. KpnI site underlined
xbaI_R_XOA17	TAAATCTAGAAGCCGTCTGACAGATTGCC	Anneals downstream of K56-2 *wabS* gene. XbaI site underlined.

Supplementary Figure S1. RimO protein sequence of Van1 versus JC1 lysogen.

```
CLUSTAL multiple sequence alignment by MUSCLE (3.8)


lys             MSKKYSIGIVSLGCPKALVDSEQIITQLRAEGYEISGTYDGADLVVVNTCGFIDEAVQES
rimO            MSQSPKVGFVSLGCPKALVDSEQIITQLRAEGYEISGTYDGADLVVVNTCGFIDEAVQES
                **:. .:*:***************************************************

lys             LDAIGEALTENGKVIVTGCLGAKSSASGSNLIEEVHPKVLAVTGPHAVGEVMQAVHSHLP
rimO            LDAIGEALTENGKVIVTGCLGAKSSASGSNLIEEVHPKVLAVTGPHAVGEVMQAVHSHLP
                ************************************************************

lys             KPHDPFVDLVPAAGIKLTPRHYAYLKISEGCNHRCTFCIIPSMRGDLVSRPVAEVMLEAE
rimO            KPHDPFVDLVPAAGIKLTPRHYAYLKISEGCNHRCTFCIIPSMRGDLVSRPVAEVMLEAE
                ************************************************************

lys             NLFKSGVKELLVISQDTSAYGVDVKYRTGFWNGKPIKTRMTDLVAALGELAAQYGAWVRL
rimO            NLFKSGVKELLVISQDTSAYGVDVKYRTGFWNGKPIKTRMTDLVAALGELAAQYGAWVRL
                ************************************************************

lys             HYVYPYPSVDEVIPLMAEGPFKGHVLPYLDVPFQHAHPEVLKRMKRPANAEKVLERVQKW
rimO            HYVYPYPSVDEVIPLMAEGPFKGHVLPYLDVPFQHAHPEVLKRMKRPANAEKVLERVQKW
                ************************************************************

lys             REICPDLTIRSTFIAGFPGETEEQFETLLDFVREAELDRVGCFAYSPVEGATANDLDGAL
rimO            REICPDLTIRSTFIAGFPGETEEQFETLLDFVREAELDRVGCFAYSPVEGATANDLDGAL
                ************************************************************

lys             PDEVREERRARFMEVAEEVSANRMQRKVGKTLKVLIDEVGEEGGIGRTAADAPEIDGVVY
rimO            PDEVREERRARFMEVAEEVSANRMQRKVGKTLKVLIDEVGEEGGIGRTAADAPEIDGVVY
                ************************************************************

lys             VEPAAKASKRYKVGDFVSVKITGADGHDLWGEV
rimO            VEPAAKASKRYKVGDFVSVKITGADGHDLWGEV
                *********************************
```
